# Metabolic GWAS-based dissection of genetic basis underlying nutrient quality variation and domestication of cassava storage root

**DOI:** 10.1186/s13059-023-03137-y

**Published:** 2023-12-14

**Authors:** Zehong Ding, Lili Fu, Bin Wang, Jianqiu Ye, Wenjun Ou, Yan Yan, Meiying Li, Liwang Zeng, Xuekui Dong, Weiwei Tie, Xiaoxue Ye, Jinghao Yang, Zhengnan Xie, Yu Wang, Jianchun Guo, Songbi Chen, Xinhui Xiao, Zhongqing Wan, Feifei An, Jiaming Zhang, Ming Peng, Jie Luo, Kaimian Li, Wei Hu

**Affiliations:** 1 National Key Laboratory for Tropical Crop Breeding, Key Laboratory of Biology and Genetic Resources of Tropical Crops, Institute of Tropical Bioscience and Biotechnology, Sanya Research Institute of Chinese Academy of Tropical Agricultural Sciences, Haikou, China; 2 Wuhan Metware Biotechnology Co., Ltd, Wuhan, China; 3 Tropical Crops Genetic Resources Institute, Chinese Academy of Tropical Agricultural Sciences, Haikou, China; 4 Hainan Key Laboratory for Protection and Utilization of Tropical Bioresources, Hainan Institute for Tropical Agricultural Resources, Chinese Academy of Tropical Agricultural Sciences, Haikou, China; 5 Hainan Yazhou Bay Seed Laboratory, Sanya, China; 6 Institute of Scientific and Technical Information, Chinese Academy of Tropical Agricultural Sciences, Haikou, China; 7 Wuhan Healthcare Metabolic Biotechnology Co., Ltd, Wuhan, China; 8 Sanya Nanfan Research Institute of Hainan University, Sanya, 572025 China

**Keywords:** Cassava, Metabolic profiling, Genome-wide association study, Natural variation, Nutrient quality, Domestication

## Abstract

**Background:**

Metabolites play critical roles in regulating nutritional qualities of plants, thereby influencing their consumption and human health. However, the genetic basis underlying the metabolite-based nutrient quality and domestication of root and tuber crops remain largely unknown.

**Results:**

We report a comprehensive study combining metabolic and phenotypic genome-wide association studies to dissect the genetic basis of metabolites in the storage root (SR) of cassava. We quantify 2,980 metabolic features in 299 cultivated cassava accessions. We detect 18,218 significant marker-metabolite associations via metabolic genome-wide association mapping and identify 12 candidate genes responsible for the levels of metabolites that are of potential nutritional importance. *Me3GT*, *MeMYB4*, and *UGT85K4*/*UGT85K5*, which are involved in flavone, anthocyanin, and cyanogenic glucoside metabolism, respectively, are functionally validated through in vitro enzyme assays and in vivo gene silencing analyses. We identify a cluster of cyanogenic glucoside biosynthesis genes, among which *CYP79D1*, *CYP71E7b*, and *UGT85K5* are highly co-expressed and their allelic combination contributes to low linamarin content. We find *MeMYB4* is responsible for variations in cyanidin 3-O-glucoside and delphinidin 3-O-rutinoside contents, thus controlling SR endothelium color. We find human selection affects quercetin 3-O-glucoside content and SR weight per plant. The candidate gene *MeFLS1* is subject to selection during cassava domestication, leading to decreased quercetin 3-O-glucoside content and thus increased SR weight per plant.

**Conclusions:**

These findings reveal the genetic basis of cassava SR metabolome variation, establish a linkage between metabolites and agronomic traits, and offer useful resources for genetically improving the nutrition of cassava and other root crops.

**Supplementary Information:**

The online version contains supplementary material available at 10.1186/s13059-023-03137-y.

## Background

Metabolites not only play critical roles in the growth and development of plants to cope with various environments but also provide essential resources for human health as foods, nutrients, and medicines [1]. In addition, metabolites are a bridge between the genome and agricultural traits and can provide unique insights into the metabolic consequences of crop domestication and improvement [2, 3]. Exploring metabolite diversity and its underlying genetic variation is thus of fundamental significance for crop breeding and germplasm conservation [4]. Combining metabolomics and other omics profiling provides an effective approach for identification of gene function and elucidation of metabolic pathways [3, 5].

Root and tuber crops (such as cassava, potato, and sweet potato) are important for human nutrition due to their high carbohydrate contents and varying levels of proteins and vitamins [6]. In recent years, although many advances have been made to characterize their genomes and agronomically important traits [7–10], the genetic bases underlying the metabolic variation in root and tuber crops are still largely unknown. Cassava (*Manihot esculenta*), a vital root crop in tropical and subtropical regions, serves as a source of nourishment for over 800 million people worldwide [11]. The cassava storage root (SR) is rich in starch, but relatively deficient in fats, proteins, minerals, and micronutrients [12, 13]. Metabolites including cyanogenic glucosides (CGs), anthocyanins, and flavonoids are key components of cassava SR quality that affect both its eating and nutritive qualities for humans [11, 14, 15]. Therefore, understanding the genetic and biochemical bases of metabolites among diverse cassava accessions will provide useful insights for breeding elite cultivars with enhanced nutrition (especially for regions in which malnutrition is widespread) and will offer valuable references for related studies in other root and tuber crops.

Metabolic genome-wide association studies (mGWAS) have been widely used to decipher the genetic basis of metabolite biosynthesis and regulation in crops including rice [5, 16], maize [17], and wheat [18, 19]. Combining mGWAS with phenotypic GWAS (pGWAS) has allowed rapid identification of candidate genes and potential networks underlying specific metabolites and has also provided evidences of metabotype-phenotype linkages [1]. Although many achievements have been made in cereals, no such comprehensive mGWAS studies have been performed in root crops such as cassava to establish possible connections between metabolites and traits, which greatly limited the progress of genetic improvement for enhanced nutrition.

Domestication has been of great importance for increasing crop yields and quality. The effects of domestication can be assessed not only at the genome and transcriptome level but also at the metabolomic level because amino acids, phenolics, organic acids, flavonoids, and carbohydrate metabolites have changed concurrent with the domestication of major cereals and fruits [2]. However, such metabolite changes and their effects on agronomic traits during cassava domestication have not yet been investigated at the metabolomic level.

Here, we describe our comprehensive metabolic profiling of 299 representative cultivated cassava accessions together with mGWAS and pGWAS to dissect the genetic bases of their metabolic diversity. We further identified 538 metabolites whose contents were likely affected during cassava domestication and further demonstrated that *MeFLS1* underwent selection resulting in increased quercetin 3-O-glucoside content. Altogether, our results identified key loci underlying the variation in metabolite contents related to SR nutrition and quality and laid a foundation for future metabolomics-assisted cassava breeding.

## Results

### Metabolic profiling of cassava cultivars

In total, 2980 metabolites were determined in SR of 299 cassava cultivars (Additional file 2: Table S1). Of which, 489 metabolites were annotated while the remaining 2491 were unknown (Additional file 2: Table S2 and Table S3). The three most abundant metabolite classes were flavones, amino acid (AA) derivatives, and lipids and then followed by organic acids, nucleotide derivates, hydroxycinnamoyl derivatives, phenolic acids, catechin derivatives, carbohydrates, anthocyanins, alkaloids, vitamins, coumarin derivatives, and cyanogenic glycosides (CGs). These results revealed the presence of a wide array of important metabolic pathways in cassava (Additional file 1: Fig. S1A). Among the 489 annotated metabolites, 431 (88%) showed significant (*r* > 0.25 and *P* < 0.01) correlations between the two biological replicates (Additional file 1: Fig. S1B), suggesting a stable repeatability.

**Fig. 1:**
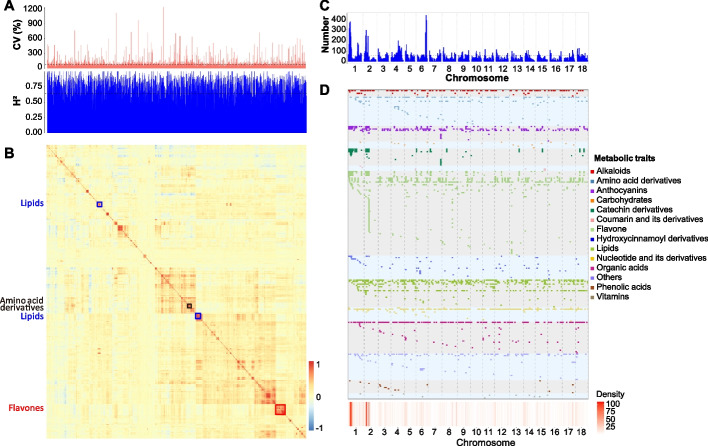
Metabolic profiling and mGWAS signal distribution. **A** Distribution of coefficient of variation (CV) and broad-sense heritability (*H*^2^) for the metabolites detected in the GWAS population. The horizontal dashed lines indicate the means of CV and H^2^, respectively. **B** Heatmap of metabolites based on their pairwise Pearson’s correlations. Positive and negative correlations are indicated in red and blue, respectively. **C** Chromosomal distribution of detected mGWAS signals. The horizontal dashed line indicates the threshold (determined by 1000 permutations) of hotspots, represented as the number of mGWAS signals within an interval of 100-kb. **D** Chromosomal distribution of mGWAS of 489 known metabolites. Each row represents the mGWAS results for a single metabolite. Metabolites from distinct chemical groups are indicated in different colors. The heatmap at the bottom indicates the density of mGWAS within each 100-kb interval across the genome

The majority (74.0%) of these metabolites showed broad-sense heritability (*H*^2^) greater than 0.5, and 25.7% displayed heritability greater than 0.8 (Fig. 1A), indicating a high genetic contribution to these metabolites. In addition, the coefficients of variation for 64.3% of these metabolites were >50% (Fig. 1A), with flavones (165.3%) and CGs (31.6%) showing the highest and lowest values, respectively. Pairwise Pearson’s correlations were calculated to elucidate the links among metabolic pathways, revealing a higher proportion of positive than negative correlations (Fig. 1B). Specifically, most flavones and AA derivatives were tightly associated in one metabolite cluster, respectively, and lipids were grouped in two major clusters (Fig. 1B and Additional file 1: Fig. S2). These highly correlated metabolites tended to be of similar structure (e.g., kaempferol 3-O-glucoside and kaempferol 3-O-robinobioside) or located on the same biochemical pathway (e.g., L-valine and L-isoleucine for CG biosynthesis, and cyanidin 3-O-glucoside and delphinidin 3-O-rutinoside for anthocyanin biosynthesis), supporting their involvement in similar physiological processes.

### Functional interpretation of cassava mGWAS

The whole genome re-sequencing of 299 cassava cultivars was performed by our previously study [10], yielding a total of 1,155,988 high-confidence SNPs that were subsequently used for mGWAS analysis. We identified 10,666 and 9848 lead SNPs corresponding to 1515 and 1576 metabolites in replicate 1 and replicate 2 (Additional file 2: Table. S4 and Table. S5), respectively, with 2263 lead SNPs and 334 metabolites identified both in these two replicates. These SNPs explained 7.3–65.8% and 8.5–62.0% of the observed metabolic variance, with a median value of 12.1% and 12.4%, respectively. Approximately 58.1% (1,731/2,980) of the detected metabolites were associated with at least one SNP, with an average of 10.5 associations per metabolite. The full list of significant mGWAS associations is summarized in Additional file 2: Table S6, as a useful resource for further validation.

The significant loci were not randomly distributed across the 18 cassava chromosomes (Fig. 1C), indicating an enrichment of major genes controlling the levels of multiple metabolites in a few regions. In total, 158 mGWAS hotspots were identified. Specifically, anthocyanins (cyanidin 3-O-glucoside and delphinidin 3-O-rutinoside), catechin derivatives (catechin gallate and epicatechin gallate), and flavones (naringenin 7-O-rutinoside, apigenin 5-O-glucoside, and genistein 7-O-glucoside) related hotspots were found on Chr1: 3.06–10.51 and Chr2: 4.38–7.51 Mb (Fig. 1D). The candidate genes and possible causative SNPs underlying variation in these metabolites were identified as follows.

Coumaroylquinic acid is one of chlorogenic acids that act as antioxidants in plants and protect against degenerative, age-related diseases in humans [20]. Sc06g003480 near a significant signal (SNP 6:10042730) associated with 5-O-p-coumaroylquinic acid encodes a hydroxycinnamoyl-CoA shikimate/quinate hydroxycinnamoyl transferase (*MeHCT*), a key enzyme involved in chlorogenic acid biosynthesis [20]. A nonsynonymous mutation SNP 6:9924983 (D406A) in the exon 2 is associated with 5-O-p-coumaroylquinic acid content and may alter the 3D structure of MeHCT protein, suggesting that *MeHCT* was the responsive gene with SNP 6:9924983 being the candidate polymorphism for the levels of 5-O-p-coumaroylquinic acid (Additional file 1: Fig. S3 and Additional file 2: Table S7).

Flavones are also effective antioxidants that can trap free radicals through redox-dependent pathways [21]. A strong mGWAS signal (SNP 16:657382) associated with myricetin 3-O-galactoside content harbors Sc16g000640 (*MeANR*), which encodes an anthocyanidin reductase that functions downstream of myricetin in the anthocyanin biosynthesis pathway [22]. A nonsynonymous mutation SNP 16:760436 (E108G) in the exon 2 is associated with myricetin 3-O-galactoside content and might alter the 3D structure of the MeANR protein, suggesting *MeANR* as a candidate gene for this metabolite (Additional file 1: Fig. S4). Similarly, Sc02g014570 (encoding a UDP-glucosyl transferase) has been tentatively assigned as a candidate gene underlying the levels of dihydrokaempferol (Additional file 1: Fig. S5). Sc02g007770 (encoding an isocitrate dehydrogenase) and Sc02g007890 (encoding a glutathione S-transferase) have been assigned as the candidates for the levels of naringenin 7-O-rutinoside and genistein 7-O-glucoside, respectively (Additional file 2: Table S7).

**Fig. 2:**
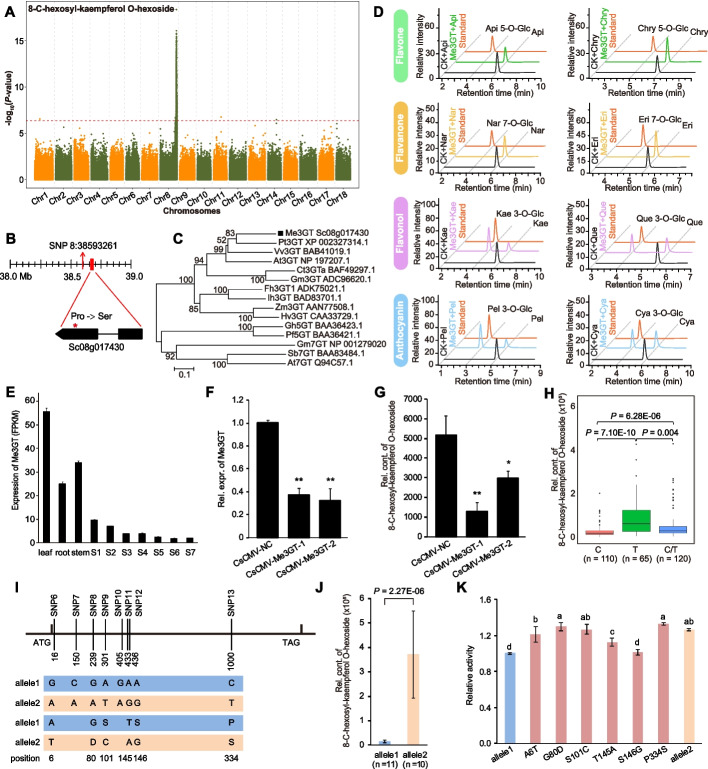
Functional annotation of genes responsible for variations in 8-C-hexosyl-kaempferol O-hexoside content. **A** Manhattan plot displaying the GWAS results for 8-C-hexosyl-kaempferol O-hexoside content. **B** Gene model of Sc08g017430 (*Me3GT*) located 87.2 kb from the lead SNP 8:38593261, which is indicated by a vertical arrow. The causative SNP 8:38681061, which is located in exon 2 of *Me3GT*, is indicated by a red star. **C** Phylogenetic tree of *Me3GT* and glucosyltransferase genes from other species. Bootstrap values are indicated at each node. **D** HPLC chromatograms of the reaction products of Me3GT with UDP-D-xylose and either apigenin 5-O-glucoside, chrysin-5-O-glucoside, naringenin 7-O-glucoside, eriodictyol 7-O-glucoside, kaempferol 3-O-glucoside, quercetin 3-O-glucoside, pelargonidin-3-O-glucoside, or cyanidin-3-O-glucoside. **E** Expression level of *Me3GT* in leaf, root, stem, and among seven developmental stages (S1–S7) of storage root. **F** Reduced transcript expression of *Me3GT* in *Me3GT*-silenced cassava plants (CsCMV-Me3GT-1 and CsCMV-Me3GT-2) compared with the control (CsCMV-NC) in leaf. Bars indicate mean ± SD, *n* = 3. **G** Reduced levels of 8-C-hexosyl-kaempferol O-hexoside in *Me3GT*-silenced cassava plants (CsCMV-Me3GT-1 and CsCMV-Me3GT-2) compared with the control (CsCMV-NC) in leaf. Bars indicate mean ± SD, *n* = 3. **H** Boxplot displaying the relative content of 8-C-hexosyl-kaempferol O-hexoside based on the causative SNP 8:38681061. **I** SNPs identified in the coding sequence of *Me3GT*. The two shaded rows above are nucleotide polymorphisms of allele1 and allele2, while the two shaded rows below are the corresponding amino acids. The SNPs (SNP6-SNP13) are corresponding to those in Additional file 2: Table. S8. **J** Relative contents of 8-C-hexosyl-kaempferol O-hexoside between allele1 and allele2. **K** Enzymatic activity among six mutants (A6T, G80D, S101C, T145A, S146G, P334S) and allele1 and allele2. Different letters indicate significant differences at *P* < 0.05 based on Duncan’s multiple range tests. Bars indicate mean ± SD, *n* = 3

Kaempferol derivatives play important roles in fruit quality and appearance [23]. A strong signal (SNP 8:38593261) associated with 8-C-hexosyl-kaempferol O-hexoside (Fig. 2A-B) harbors Sc08g017430, which encodes a UDP-glucosyl transferase (*Me3GT*) involved in glucosylation of flavonols such as kaempferol [24]. *Me3GT* showed high sequence similarity with 3GTs from *Populus trichocarpa* and *Vitis vinifera* (Fig. 2C). Our in vitro enzymatic activity assay confirmed that *Me3GT* has 3-O-glucosyltransferase activity, as it only catalyzed the glucosyltransferation reaction to generate kaempferol 3-O-glucoside, quercetin 3-O-glucoside, pelargonidin-3-O-glucoside, or cyanidin-3-O-glucoside, but did not produce apigenin 5-O-glucoside, chrysin-5-O-glucoside, naringenin 7-O-glucoside, or eriodictyol 7-O-glucoside (Fig. 2D). *Me3GT* exhibited higher transcript abundances in leaf and stem than that in SR (Fig. 2E). Transient silencing of *Me3GT* resulted in dramatically decreases in 8-C-hexosyl-kaempferol O-hexoside contents (Fig. 2F-G). Further analysis revealed that SNP 8:38681061 on the exon 2 of *Me3GT* resulted in a nonsynonymous mutation from Pro to Ser (P334S) and was significantly associated with the levels of 8-C-hexosyl-kaempferol O-hexoside (Fig. 2B and Fig. 2H). PCR-based resequencing of *Me3GT* verified this variation and further identified 12 additional SNPs (including five at the promoter area, two synonymous and five nonsynonymous mutations) showing highly significant associations with the levels of 8-C-hexosyl-kaempferol O-hexoside (Additional file 2: Table S8). The SNPs in the coding region formed two alleles (allele1 and allele2) that displayed significant differences in 8-C-hexosyl-kaempferol O-hexoside content and enzymatic activity (Fig. 2I-K). Site-directed mutagenesis showed that five of the six nonsynonymous mutations (except S146G) strongly affected the enzymatic activity of Me3GT (Fig. 2K), suggesting that they are functional SNPs of this gene. Together, these results indicate *Me3GT* as the gene responsible for 8-C-hexosyl-kaempferol O-hexoside content in cassava roots.

### A CG biosynthesis-related gene cluster influencing linamarin content

**Fig. 3:**
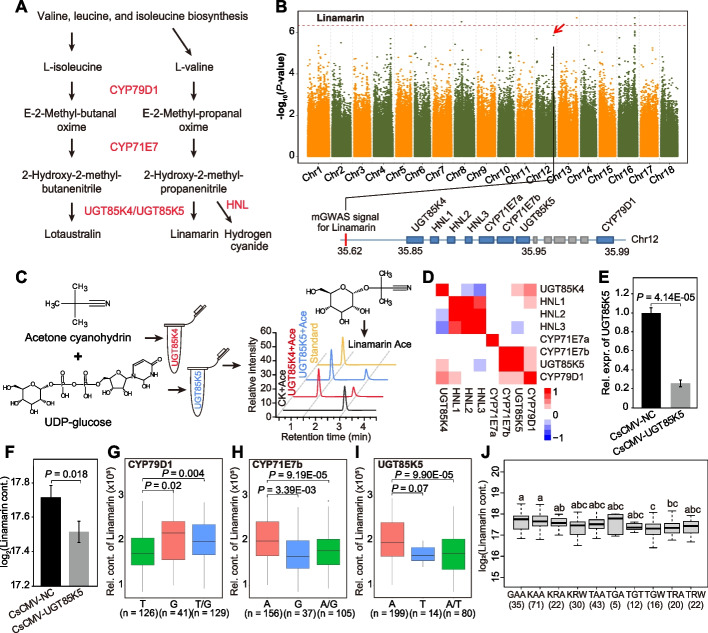
Functional annotation of genes responsible for CG metabolism. **A** Schematic representation of the CG biosynthesis pathway, in which the key enzymes are highlighted in red. **B** Manhattan plot displaying the GWAS results for linamarin content. The lower panel shows the location of five CG biosynthesis genes and three HNLs in a 140-kb region. **C** HPLC chromatograms of the products of the reactions of UGT85K4 and UGT85K5, respectively, with UDP-glucose and acetone cyanohydrin. **D** Expression correlation of CG metabolism genes in SR of 22 cultivated cassava accessions. **E** Reduced transcript expression of *UGT85K5* in *UGT85K5*-silenced cassava plants (CsCMV-UGT85K5) compared with the control (CsCMV-NC) in leaf. Bars indicate mean ± SD, *n* = 3. **F** Reduced levels of linamarin in *UGT85K5*-silenced cassava plants (CsCMV-UGT85K5) compared with the control (CsCMV-NC) in leaf. Bars indicate mean ± SD, *n* = 3. **G–I** Boxplot displaying the relative contents of linamarin based on the causative SNPs 12:35990792 (for *CYP79D1*), 12:35945868 (for *CYP71E7b*), and 12:35953769 (for *UGT85K5*), respectively. **J** The relative content of linamarin based on the allelic combination of causative SNPs 12:35990792 (for *CYP79D1*), 12:35945868 (for *CYP71E7b*), and 12:35953769 (for *UGT85K5*). Different letters indicate significant differences at *P* < 0.05 based on Duncan’s multiple range tests

CG content is a quality trait that adversely influences the edibility of cassava SR [11]. The two major forms of CG in cassava SR, linamarin and lotaustralin, are synthesized from L-valine and L-isoleucine, respectively, by the same set of enzymes including CYP79D1, CYP71E7, and UGT85K4/UGT85K5 [25]. In addition, hydroxynitrile lyase (HNL) is responsible for the generation of hydrogen cyanide from 2-hydroxy-2-methyl-propanenitrile (Fig. 3A). A clear mGWAS signal was observed for linamarin content on Chr16:32817362. Several signals displaying weakly associations with linamarin content were also observed on Chr5, 8, 12, and 13 (Fig. 3B). Among these, the signal (SNP 12:35620552) on Chr12 harbors a gene cluster containing five CG biosynthesis genes (*CYP79D1*, *CYP71E7a*, *CYP71E7b*, *UGT85K4*, and *UGT85K5*) and three tandemly repeated HNL-encoding genes (*HNL1*–*3*) in a 140-kb region that were identified through BLASTP search by querying with previously known homolog genes [26].

*UGT85K5* shows high sequence identity with *UGT85K4* and both can catalyze the glycosylation of acetone cyanohydrin to produce linamarin (Fig. 3C and Additional file 1: Fig. S6). However, *UGT85K5*, rather than *UGT85K4*, was highly co-expressed with *CYP79D1* and *CYP71E7b* (Fig. 3D and Additional file 1: Fig. S7), implying their common involvement in CG biosynthesis. Transient silencing of *UGT85K5* resulted in significantly decreases in linamarin contents (Fig. 3E-F). The SNP variant Chr12:35990792 in the 3’UTR of *CYP79D1* was associated with linamarin content, as cassava accessions harboring the T-allele showed significantly lower linamarin content than did the accessions carrying the G-allele (Fig. 3G). Additionally, the SNP Chr12:35945868 in the intergenic region of *CYP71E7b* and the SNP Chr12:35953769 in the downstream of *UGT85K5* were also associated with linamarin content. Linamarin content was significantly higher in accessions carrying the A-allele than in accessions carrying the G-allele or T-allele of these two genes, respectively (Fig. 3H-I). Moreover, cassava accessions carrying a TGW allele combination (i.e., T-allele for *CYP79D1*, G-allele for *CYP71E7b* and A/T-allele for *UGT85K5*) showed the lowest content of linamarin (Fig. 3J). PCR-based resequencing was performed to investigate more functional variations of *CYP79D1*, *CYP71E7b*, and *UGT85K5*. Highly significant associations were identified between three SNPs (SNP1-SNP3) in the promoter of *CYP79D1* and the levels of linamarin (Additional file 2: Table S9). However, they are not likely the causative SNPs since no significant (*P* = 0.84) correlation was observed between the expression of *CYP79D1* and the levels of linamarin across 22 cassava cultivars. Three SNPs (SNP14-SNP16, of which SNP16 is the causative SNP Chr12:35953769) in the 3’UTR of *UGT85K5* showed highly significant associations with the levels of linamarin (Additional file 2: Table S10), while no significant associations were observed between the SNPs of *CYP71E7b* and the levels of linamarin (Additional file 2: Table S11).

### Anthocyanins determine cassava SR endothelium color

**Fig. 4:**
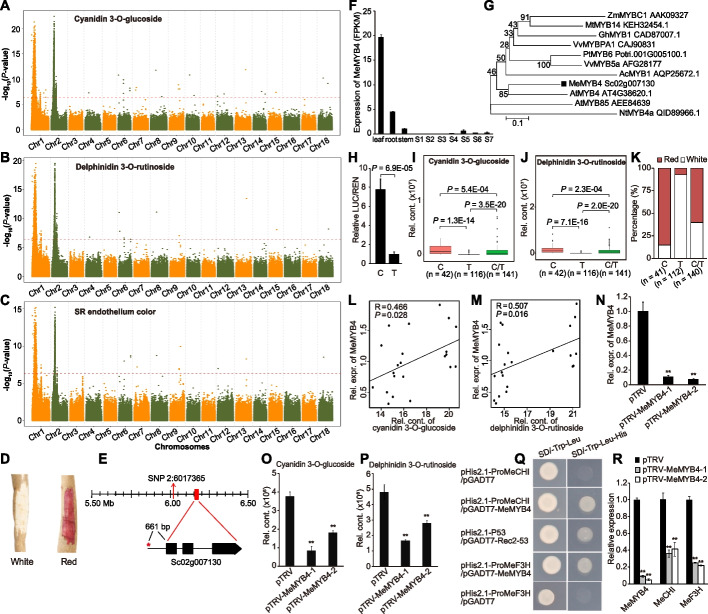
Functional annotation of genes responsible for variation in anthocyanin contents and SR endothelium color. **A-B** Manhattan plot displaying the GWAS results of cyanidin 3-O-glucoside and delphinidin 3-O-rutinoside. **C** Manhattan plot displaying the GWAS results for SR endothelium color. **D** Phenotypes of cassava SR with white or red endothelium. **E** Gene model of Sc02g007130 (*MeMYB4*). The lead SNP 2:6017365 is indicated by a vertical arrow. The causative SNP 2:6158256, which located in the promoter of *MeMYB4*, is indicated by a red star. **F** Expression level of *MeMYB4* in leaf, root, stem, and among seven developmental stages (S1–S7) of storage root. **G** Phylogenetic tree of *MeMYB4* and MYB genes from other species. Bootstrap values are indicated at each node. **H** Comparison of activities between C-containing and T-containing promoter regions (1000 bp upstream of ATG) in dual luciferase assay. Each sample contains seven biological replicates. **I-J** Boxplot displaying the relative contents of cyanidin 3-O-glucoside and delphinidin 3-O-rutinoside based on the single likely causative SNP 2:6158256. **K** The percentage of SR endothelium color attributable to the causative SNP 2:6158256. **L-M** Pearson correlations between the expression of *MeMYB4* and the content of cyanidin 3-O-glucoside and delphinidin 3-O-rutinoside in SR of 22 cassava cultivars, respectively. **N** Reduced transcript expression of *MeMYB4* in *MeMYB4*-silenced cassava plants (pTRV-MeMYB4-1 and pTRV-MeMYB4-2) compared with the control (pTRV) in leaf. Bars indicate mean ± SD, *n* = 3. **O-P** Reduced levels of cyanidin 3-O-glucoside and delphinidin 3-O-rutinoside in *MeMYB4*-silenced cassava plants (pTRV-MeMYB4-1 and pTRV-MeMYB4-2) compared with the control (pTRV) in leaf. Bars indicate mean ± SD, *n* = 3. **Q** Growth of yeast cells co-transformed with pHis2.1-ProMeCHI/pGADT7-MeMYB4, pHis2.1-ProMeF3H/pGADT7-MeMYB4, or the positive control (pHis2.1-P53/pGADT7-Rec2-53). **R** Reduced transcripts of *MeCHI* and *MeF3H* in *MeMYB4*-silenced cassava plants (pTRV-MeMYB4-1 and pTRV-MeMYB4-2) compared with the control (pTRV) in leaf. Bars indicate mean ± SD, *n* = 3. Asterisk symbols (**) in this figure indicate a significant difference at *P* < 0.01 by the Student’s *t*-test

Anthocyanins are responsible for some of the red coloration and nutritional qualities of plant-derived foods [27]. Cassava SR with red endothelium is highly valued for its appearance and is therefore popular among consumers. The metabolites cyanidin 3-O-glucoside and delphinidin 3-O-rutinoside involved in the anthocyanin pathway exhibit highly correlated contents (*Cor* = 0.94, *P* < 2.2e-16) in cassava SR. A total of two mGWAS signals were detected for the contents of these metabolites on Chr1-9.13 Mb and Chr2-6.09 Mb, respectively (Fig. 4A-B). Moreover, these two signals were co-located with the GWAS signals for SR endothelium color (Fig. 4C-D). These signals both harbor the candidate Sc02g007130 (*MeMYB4*) encoding an ortholog of *AtMYB4*, which is involved in anthocyanin biosynthesis [28]. A possible causative SNP 2:6158256 in the 5’UTR of *MeMYB4* is significantly associated with cyanidin 3-O-glucoside and delphinidin 3-O-rutinoside contents and also with SR endothelium color (Fig. 4E-G). PCR-based resequencing verified this variation as well as these associations in 22 cassava accessions (Additional file 2: Table S12). The dual luciferase assay found that the promoter region of *MeMYB4* carrying C-allele had higher activity than those carrying T-allele, indicating that this allelic variation affects *MeMYB4* expression (Fig. 4H). Cassava accessions carrying the C-allele showed higher metabolic levels of cyanidin 3-O-glucoside and delphinidin 3-O-rutinoside than did those carrying the T-allele (Fig. 4I-J) and accordingly included a higher percentage of cultivars with red SR endothelium (Fig. 4K). Expression of *MeMYB4* was significantly (*P* < 0.05) correlated with the metabolite levels of both cyanidin 3-O-glucoside and delphinidin 3-O-rutinoside (Fig. 4L-M). Transient silencing of *MeMYB4* resulted in dramatically decreases in cyanidin 3-O-glucoside and delphinidin 3-O-rutinoside contents (Fig. 4N-P). Further, yeast one-hybrid assays revealed that MeMYB4 protein could bind directly to the promoter regions of *MeCHI* (Sc07g012880) and *MeF3H* (Sc02g010090), which encode two key enzymes involved in anthocyanin biosynthesis (Fig. 4Q). Transient silencing of *MeMYB4* also resulted in significantly decreases in the expression of *MeCHI* and *MeF3H* (Fig. 4R). Collectively, these results strongly indicate the positive control of *MeMYB4* in cyanidin 3-O-glucoside and delphinidin 3-O-rutinoside as well as red endothelium in SR through its interaction with *MeCHI* and *MeF3H*.

### Effects of domestication on metabolite contents and the negative roles of quercetin 3-O-glucoside in cassava yield

Due to the rapid modification of agriculturally important traits by humans, metabolite contents of the edible portions of crop species have also been strongly affected during domestication. To provide insights into the metabolic changes that have taken place during cassava domestication, metabolite profiles were compared between 299 cultivated and five representative wild cassava accessions (Additional file 1: Fig. S8), resulting in a total of 538 differentially accumulated metabolites (DAMs). Among the 132 of these metabolites that were annotated, flavones and lipids have changed the most with domestication. Compared with wild accessions, the contents of the majority of AA derivatives were higher in cultivars, whereas most flavones, lipids, phenolic acids, hydroxycinnamoyl derivatives, and nucleotide derivates were lower, with average changes as low as 0.14-, 0.08-, 0.06-, 0.07-, and 0.18-fold, respectively (Additional file 2: Table S13). Domestication also caused significant increases in SR weight [10], guiding us to investigate the contribution of metabolites to SR weight.

**Fig. 5:**
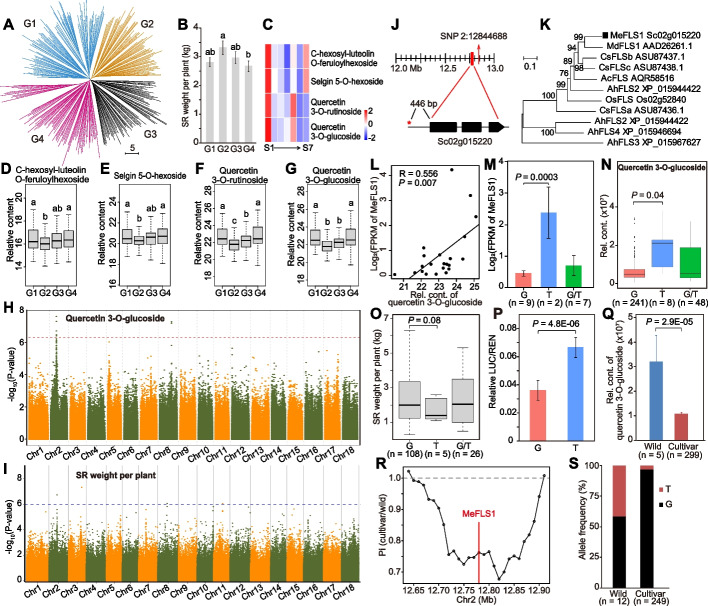
Functional annotation of genes responsible for variation in quercetin 3-O-glucoside content and SR weight per plant. **A** Clustering of four groups (G1–G4) of cultivated cassava based on the metabolite profiles in SR. **B** Differences in SR weight per plant among four groups (G1–G4) of cultivated cassava shown in **A**. Different letters indicate significant differences at *P* < 0.05 based on Duncan’s multiple range tests. **C** Relative contents of four flavones among seven developmental stages (S1–S7) of storage root in cassava cultivar SC205. **D-G** Relative contents of four flavones negatively correlated with SR weight per plant among four cassava groups (G1–G4) shown in **A**. **H** Manhattan plot displaying the GWAS results for quercetin 3-O-glucoside contents. **I** Manhattan plot displaying the GWAS results for SR weight per plant. **J** Gene model of Sc02g015220 (*MeFLS1*) located 65.6 kb from the lead SNP 2:12844688, which is indicated by a vertical arrow. The causative SNP 2:12776464, which located in the 3’UTR of *MeFLS1*, is indicated by a red star. **K** Phylogenetic tree of *MeFLS1* and FLS genes from other species. Bootstrap values are indicated at each node. **L** Pearson correlation between the expression of *MeFLS1* and the content of quercetin 3-O-glucoside in SR of 22 cassava cultivars. **M** Differences in expression of *MeFLS1* in SR attributable to the causative SNP 2:12776464. **N** Boxplot displaying the relative content of quercetin 3-O-glucoside attributable to the causative SNP 2:12776464. **O** Boxplot displaying SR weight per plant attributable to the causative SNP 2:12776464. **P** Comparison of activities between G-containing and T-containing promoter regions (1000 bp upstream of ATG) in dual luciferase assay. Each sample contains seven biological replicates. **Q** Differences in quercetin 3-O-glucoside contents between wild and cultivated cassava. The significance in (M-Q) was determined by the Student's *t*-test. **R** Nucleotide diversity (Pi) between the wild (*n* = 19) and cultivated (*n* = 299) cassava accessions around the *MeFLS1* region in 80-kb sliding windows with 10-kb steps. **S** Allele frequencies of the causative SNP 2:12776464 between wild and cultivated cassava accessions

Clustering of metabolite levels divided the cassava cultivars into four main groups that exhibited significant differences in SR weight per plant (Fig. 5A-B). Four flavones involved in SR development were significantly negatively correlated with SR weight per plant (Fig. 5C-G), suggesting a possible contribution of flavones to SR weight per plant via an influence on SR development [29]. The roles of flavones in SR weight per plant were further investigated based on the mGWAS and pGWAS co-locations, which revealed that mGWAS signals for 16 flavones were co-located with the pGWAS signal for SR weight per plant on Chr2:12.84 Mb (Fig. 5H-I and Additional file 1: Fig. S9). Among these, 14 flavones were associated with SR development (Additional file 2: Table S14), including quercetin 3-O-glucoside as one of the four flavones negatively associated with SR weight per plant (Fig. 5B and Fig. 5G). Candidate gene mining found that Sc02g015220 encoded a flavonol synthase (*MeFLS1*, Fig. 5J-K), a key enzyme involved in the production of quercetin in the flavonoid biosynthesis pathway [30]. The expression of *MeFLS1* was significantly (*P* < 0.05) correlated with the metabolite levels of quercetin 3-O-glucoside contents in SR among different cultivars (Fig. 5L). Further, a causative SNP 2:12776464 in the promoter region of *MeFLS1* might be associated with *MeFLS1* expression levels, quercetin 3-O-glucoside content, and SR weight per plant. Cassava accessions harboring the G-allele of *MeFLS1* had lower expression level (*P* = 0.0003), lower quercetin 3-O-glucoside content (*P* = 0.04), and marginally higher SR weight per plant (*P* = 0.08) than did the accessions containing the T-allele (Fig. 5M-O). The dual luciferase assay showed that the promoter region of *MeFLS1* carrying G-allele had lower activity than those carrying T-allele, suggesting that this allelic variation affects *MeFLS1* expression (Fig. 5P). Quercetin 3-O-glucoside content was significantly higher in wild accessions than in cultivars (Fig. 5Q). Nucleotide diversity (Pi) around the *MeFLS1* region was significantly lower in cultivars than in wild accessions (Fig. 5R), indicating that the *MeFLS1* region was under selection during cassava domestication. Moreover, the frequency of the G-allele was higher in cultivars compared with wild accessions (Fig. 5S). Together, these results suggested that *MeFLS1* was the gene responsible for variation in the contents of quercetin 3-O-glucoside, which might have negatively influenced SR weight per plant during cassava domestication.

## Discussion

### Metabolomics and mGWAS

Determination of metabolites is a fundamental step for studying the genetic control of their contents and thereby takes advantage of variation to improve nutritional quality of crops. The SR is the most economically valuable part of the cassava plants and contributes substantially to the diets of humans in the tropical regions. The present study identified 2980 metabolites (including 489 structurally annotated) in SR of 299 cassava cultivars (Additional file 2: Table S2 and Table S3). The number of metabolites detected here represents a considerable advance in metabolite detection compared with previous studies in cassava [14, 31–33]. Approximately 64.3% of these metabolites exhibited coefficients of variation >50%. A few specifically important metabolites such as linamarin and lotaustralin, which have major negative impacts on SR edibility, were included in our study (Additional file 2: Table S2). Similar to previous reports in rice, tomato, and wheat [34–36], metabolites such as flavones, lipids, and amino acids generally showed strong within-class correlations (Fig. 1), providing a useful resource for identifying unknown metabolites based on correlations of their contents with those of known metabolites [5]. These results will deepen our understanding of natural variation and sub-networks of metabolites in SR and suggest further exploration of how variation in the contents of these metabolites could influence cassava nutritional quality.

By combining our analysis of metabolite contents in cassava SR with 1,155,988 high-confidence SNPs, a total of 18,218 mGWAS associations were found. These associations were non-randomly distributed across the genome as previously observed in rice, maize, and wheat [34, 36, 37], suggesting that multiple metabolites can be influenced by manipulation of a small genomic region during metabolomics-assisted cassava breeding. Here, a total of 12 candidate genes were identified as associated with 11 metabolic traits (Additional file 2: Table S7). The reliability of these candidates was supported by the associations between metabolite levels and allelic variations in the candidate genes, as well as by the functional validation of their orthologs in other species. Several of the candidates we designated were then further analyzed by in vitro enzyme assays or the transient silencing due to the challenges of stable genetic transformation [38]. These data will facilitate future genetic improvement and breeding strategies in cassava.

### CG biosynthesis genes

As a major component of SR quality, CGs play a key role in plant defense but are also toxic to humans [11]. The CG concentrations in SR of cassava cultivars must be reduced to a safe level before human consumption. Therefore, improving cassava varieties with low CG and exploring the genetic architecture of CG in cassava are of considerable interest.

Through quantitative trait locus (QTL) analysis, previous studies have found several loci controlling CG content in cassava roots, but did not provide conclusive information on the genetic basis of CG variation due to limitations in available genomic resources and QTL detection power [39, 40]. More recently, Ogbonna et al. [11] performed a GWAS using multiyear trials and identified two major regions associated with CG variation on chromosomes 14 and 16, respectively. However, these studies assessed CG content as a whole and did not provide any specific information regarding the contents of linamarin and lotaustralin, two major forms of CG in cassava. In this work, the contents of linamarin and lotaustralin were assayed in 299 diverse cultivars, and four mGWAS associations were detected for linamarin while only one was detected for lotaustralin (Fig. 3B and Additional file 1: Fig. S10). This might be because linamarin is more abundant (~95% of CG) than lotaustralin in cassava SR [41, 42]. Interestingly, the association of SNP 14:6662981 with lotaustralin content was congruent with the location of Manes.14G073900 (encoding a plasma membrane ATPase) that is associated with variation in hydrogen cyanide (HCN) in cassava root [11]. The position of another HCN-associated gene Manes.16G007900 encoding a multi-antimicrobial extrusion protein [11] was also close to SNP 16:1643714, which showed a weak association (*P* = 1.8E-06) with linamarin in this study (Fig. 3B). These results strongly supported the accuracy and precision of our mGWAS associations and also suggested that Manes.16G007900 and Manes.14G073900 could be responsible for the contents of linamarin and lotaustralin, respectively, in SR.

A clear mGWAS signal was found for linamarin content at Chr16:32817362 (Fig. 3B). This locus has not been reported so far and might represent a novel genetic factor influencing linamarin in cassava. A gene cluster containing five CG biosynthesis genes and three HNLs was found in a 140-kb region near the locus Chr12:35620552 (Fig. 3B). We further observed that *CYP79D1*, *CYP71E7b*, and *UGT85K5* were highly co-expressed and that variants in these three genes were associated with low levels of linamarin content. Thus, breeding cassava varieties with decreased CG content might be feasible via genetic manipulation of *CYP79D1*, *CYP71E7b*, and *UGT85K5*.

CG content is associated with the resistance of cassava to biotic stress and its accumulation is induced by jasmonic acid (JA) [11, 43]. We examined the expression of CG biosynthesis genes in response to MeJA treatment and *Xanthomonas axonopodis pv. manihotis* (*Xam*) infection and found that *UGT85K4*, *CYP71E7b*, and *CYP79D1* were significantly induced at 6 h or 12 h after MeJA treatment (Additional file 2: Table S15). Similarly, *CYP71E7b* and *CYP79D1* were significantly induced by *Xam* infection at 7 dpi, although *UGT85K4* was depressed (Additional file 2: Table S15). However, the haplotypes of CG biosynthesis genes (*CYP79D1*, *CYP71E7b*, and *UGT85K5*) identified in this study are not significantly (*P* > 0.05) associated with the resistance of cassava bacterial blight (*Xam* infection).

CG content is an important domesticated trait in cassava SR that can be decreased by selection [44]. Our metabolic data revealed that the contents of substrates for CG biosynthesis including L-valine and L-isoleucine (as well as L-leucine) were significantly higher in cultivars than in wild accessions. Linamarin showed a reverse trend whereas lotaustralin was not significantly changed (Additional file 1: Fig. S11), suggesting that domestication might have reduced the efficiency of conversion from L-valine to linamarin. However, no significant selection signal was detected in the region of the 140-kb cluster of genes associated with CG biosynthesis, supporting another scenario that cassava domestication might have specifically targeted the regulatory rather than the structural genes involved in CG biosynthesis [11]. Further investigations are still required to clarify the domestication of CG in cassava.

### Associations between metabolic and phenotypic traits

Parallel GWAS analysis of metabolic and phenotypic traits greatly facilitate the dissection of their associations and are helpful for mining metabolites associated with the regulation of phenotypic traits [1, 37, 45]. This method is especially important for the analysis of traits whose genetic basis remains unknown, providing a valuable approach to explore candidate genes.

In this work, the mGWAS signals for two anthocyanin-related metabolites, cyanidin 3-O-glucoside and delphinidin 3-O-rutinoside, co-localized with the pGWAS signal for SR endothelium color (Fig. 4A-C), which established genetic associations between these two metabolites and SR endothelium color. These results also prompted our search for candidate genes related to anthocyanin biosynthesis in the co-localization region. *MeMYB4* was designated as a candidate gene within the signal regions because it displayed high sequence similarity to *AtMYB4*, which participated in anthocyanin biosynthesis in *Arabidopsis* [28]. Moreover, the SNP 2:6158256 within the 5’UTR of *MeMYB4* was significantly associated with cyanidin 3-O-glucoside and delphinidin 3-O-rutinoside contents as well as with SR endothelium color (Fig. 4I-K). MYB4s are usually the repressors on phenylalanine metabolism, but there still are a few reports showing that they (e.g., *NtMYB4a* and *BjMYB4*) act as activators involved in anthocyanin synthesis [46, 47]. MYB genes can transcriptionally regulate the expression of anthocyanin biosynthetic genes via the MYB-bHLH-WDR complex [48]. Therefore, we examined the role of *MeMYB4* via expression analysis and transient silencing and investigated the potential interactions of *MeMYB4* using yeast one-hybrid assays (Fig. 4L-R). From results of those experiments, we concluded that MeMYB4 interacts with the promoter of *MeCHI* and *MeF3H* to control the red SR endothelium phenotype by regulating the levels of cyanidin 3-O-glucoside and delphinidin 3-O-rutinoside. Collectively, the connections established between metabolites and phenotypes in this study should help to clarify their genetic and biochemical regulation and lead to more rational genetic improvements in cassava.

Similarly, five flavones involved in SR development were found to be negatively associated with SR weight per plant (Fig. 5C-H). This established possible links between flavones and root yield in cassava, as found in a study showing that flavones could affect root development in the host plant through modulation of distinct bacterial taxa [29]. The relationship between flavones and root yield in cassava is further supported by the co-localization of mGWAS and pGWAS signals for flavone contents and SR weight per plant on Chr2, in which *MeFLS1*, which is involved in production of quercetin in the flavonoid biosynthesis pathway, was found to be associated with both quercetin 3-O-glucoside content and SR weight [30, 49]. Although domestication has caused significant increase in cassava SR yield, the genetic mechanism underlying this artificial selection remains elusive [44, 50]. Our allelic variation and gene diversity analyses have revealed that artificial selection of *MeFLS1* contributed to the domestication of the large starchy cassava storage root through decreasing quercetin 3-O-glucoside levels. These findings will deepen our understanding of cassava domestication and provide potential future targets for selection to increase cassava yield.

To the best of our knowledge, this is the first application of mGWAS to illustrate the genetic basis of variation in metabolites in a large number of accessions in a root or tuber crop, which will shed light on the discovery of still more useful genes for the genetic improvement of these crops. In the near future, more wild accessions should be collected and sequenced to further verify the accuracy of our domestication analysis because of the limited number of wild accessions used in this work.

## Conclusions

In the present study, we quantified 2980 metabolic features in 299 cultivated cassava accessions, detected 18,218 significant marker-metabolite associations, and discovered 12 candidate genes responsible for the levels of metabolites of potential nutritional importance or associated with cassava domestication. We also identified a CG biosynthesis-related gene cluster in which *CYP79D1*, *CYP71E7b*, and *UGT85K5* are highly co-expressed and whose allelic combinations contribute to low linamarin content. Using parallel mGWAS and pGWAS, we found that *MeMYB4* is responsible for the variation in anthocyanin (i.e., cyanidin 3-O-glucoside and delphinidin 3-O-rutinoside) contents in SR and SR endothelium color and that *MeFLS1* is responsible for quercetin 3-O-glucoside content and SR weight per plant. Moreover, artificial selection acting on *MeFLS1* decreased quercetin 3-O-glucoside content and thus increased SR weight per plant during cassava domestication. Our study has provided novel insights into the genetic bases of cassava metabolome variation and established linkages between metabolites and agronomic traits in SR, which will facilitate the genetic improvement of cassava for enhanced nutrition.

## Methods

### Plant materials

The 299 cultivated and 5 representative wild cassava accessions used for metabolomic profiling in this study were taken from a germplasm collection in DanZhou (109.50°E, 19.51°N) at the Chinese Academy of Tropical Agricultural Sciences (Additional file 2: Table S1). For each plant, only one typical SR was sampled and cut into ~3-mm-thick slices from the middle of SR. Five to six slices from three different plants per accession were pooled as one biological replicate, and placed in liquid nitrogen immediately, and stored at −80°C until use [31]. In total, two biological replicates per accession were collected for metabolite profiling.

### Genome re-sequencing and SNP calling

Whole genome re-sequencing of the 299 cultivated and 5 wild cassava accessions mentioned above was performed in our previous work [10]. Re-sequencing data for 14 additional wild accessions derived from Bredeson et al. [51] and Ramu et al. [44] were also used in the present study. Raw reads with greater than 5% missing bases or with greater than 50% bases of base quality < 5 were discarded. The remaining clean reads were mapped to the cassava SC205 reference genome [9] using BWA mem v0.7.17 [52] with default parameters, and the results were further filtered using Samtools v1.9 [53] and Picard v1.94 (https://broadinstitute.github.io/picard/). After removing low mapping-quality (MQ < 20) reads, both single- and paired-end mapped reads were used to detect SNPs using the GATK toolkit v3.5 [54]. Finally, 1,155,988 high-confidence SNPs were identified and further annotated using the ANNOVAR program [55].

### Metabolite profiling

The untargeted metabolome was profiled at the Wuhan Metware Biotechnology Co., Ltd., as previously described [56]. For each sample, approximately 80 mg crushed lyophilized storage root was extracted overnight at 4 °C with 1.0 mL 70% aqueous methanol before analysis using an LC-ESI-MS/MS system (HPLC, Shim-pack UFLC SHIMADZU CBM30A system; MS, Applied Biosystems 6500 plus Q TRAP). The HPLC used a Waters ACQUITY UPLC HSS T3 C18 column (1.8 µm, 2.1 mm × 100 mm) at 40 °C with a solvent system of water (0.04 % acetic acid) and acetonitrile (0.04 % acetic acid) following a gradient program of 95:5 V/V at 0 min, 5:95 V/V at 11.0 min, 5:95 V/V at 12.0 min, 95:5 V/V at 12.1 min, and 95:5 V/V at 15.0 min, with a flow rate of 0.35 mL/min and injection volume of 2.0 μL. The ESI source operation parameters were used as following: ion source, turbo spray; source temperature, 500 °C; ion spray voltage, (+) 5500 V and (−) 4500V; ion source gas I (GSI), gas II (GSII), and curtain gas (CUR) were set at 55, 60, and 35 psi, respectively; the collision gas (CAD) was medium. Instrument tuning and mass calibration were performed with 10 and 100 μmol/L polypropylene glycol solutions in linear ion trap (LIT) and triple quadrupole (QQQ) modes, respectively. QQQ scans were acquired as MRM experiments with collision gas (nitrogen) set to 5 psi. A specific set of MRM transitions were monitored for each period according to the metabolites eluted within this period.

### Metabolite-based genome-wide association analyses

The biallelic SNPs with minor allele frequency (MAF) > 0.05 and missing call frequency (MCF) < 0.1 were selected for mGWAS using a compressed mixed linear model (cMLM) provided by the TASSEL program [57]. Population structure was calculated by the ADMIXTURE software [58], kinship was calculated by the TASSEL program [57], and they were incorporated in a fixed and random effect MLM to control false positives. Broad-sense heritability (*H*^2^) was calculated by using the following formula : *H*^2^ = var(G)/var(G)+var(E), in which var(G) and var(E) are the genetic and environmental variances, respectively [57]. The significant threshold of mGWAS was set to *P* ≤ 4.43e-07 after Bonferroni correction. Manhattan graphs were drawn by R (https://www.r-project.org/) in order to visualize the significant associated area and model effect. The adjacent significant SNPs (with a minimum of five) whose distance is less than 1 Mb were regarded as a significant locus, and the most significant SNP in this locus was defined as a lead SNP.

### Phenotypic genome-wide association analyses

A total of 337 accessions were planted in March 2013 and 2016 and harvested in February 2014 and 2017, respectively, in Danzhou, Hainan Province. Each accession was cultivated in single rows of eight plants with 0.8 m distance between two plants. SR endothelial color was visually evaluated and classified as either red or white. SR weight per plant was calculated as the average of eight plants. Phenotypic genome-wide association analyses were performed as previously described [10].

### Mining of candidate genes and causative SNPs

Combined biological and bioinformatics approaches [5] were used to mine candidate genes by (1) looking for a protein or protein cluster that is biochemically relevant to the associated metabolites encoded at these loci; (2) performing cluster analysis of candidate genes relative to homologous genes with known function; and (3) cross-referencing with statistical results of Pearson correlation between the metabolic levels and candidate gene expression in different tissues or cultivars. The SNPs located within, 2 kb upstream, or 2 kb downstream of the candidate genes were used to examine their associations with the corresponding metabolic traits, and the significant SNPs were considered as possible causative SNPs underlying metabolites.

### Identification of metabolites associated with domestication

Five of the 299 cultivars were randomly chosen and compared with the 5 wild accessions to identify differentially accumulated metabolites (DAMs), setting the variable importance in projection (VIP) ≥1 and |fold-change| ≥1.5 [31]. This process was repeated 10 times because of the small sample size. If a metabolite was found to have differentially accumulated in all of the 10 comparisons, it was considered associated with cassava domestication.

Phylogeny of wild and cultivated cassava accessions was generated by the neighbor-joining tree method with genome-wide SNPs [10]. Nucleotide diversity (Pi) between the wild and cultivated cassava accessions were calculated by VCFtools [59] software in 80-kb sliding windows with 10-kb steps.

### Phylogenetic analysis

The amino acid sequences of reported genes (including glucosyltransferases, flavonol synthases, and MYB transcription factors) were obtained from the NCBI database (http://www.ncbi.nlm.nih.gov/) according to their accession numbers. The information of candidate genes in this work was obtained from our recently published genome of SC205 [9]. Sequence alignments were performed using ClustalX, and the neighbor-joining tree was constructed using MEGA6 with default parameters. The reliability of the phylogenetic tree was evaluated by a bootstrap test with 1000 replicates.

The metabolic data generated from 299 cultivated cassava accessions were log2-transformed to construct a neighbor-joining tree for demonstration of the cassava population structure. The neighbor-joining tree was constructed based on the pairwise population distance by the software PHYLIP v3.69 and then visualized by the software MEGA6.

### Detection of mGWAS signal hotspots

As previously described [5], a permutation test was performed to assess the statistical significance of the deviation of the observed signal distribution from a uniform distribution. In the permutation, all signals were randomly assigned into genomic regions for each 1-Mb interval, and the number of signals for each interval was recorded. After a 1000-permutation test, the value of significant (*P* < 0.01), the number of mGWAS signals per Mb would be 60, and the intervals with a larger number of mGWAS signals were regarded as hotspots.

### RNA-seq and data analysis

To explore the expression patterns of candidate genes, samples collected from different tissues (leaf, root, and stem) as well as seven developmental stages (S1–S7) of SR were analyzed in cultivar SC205 using RNA-seq. Cassava SRs were harvested every 40 days from 100 days after planting to 340 days after planting for a total of seven time-points, referred to as S1–S7 [31]. The SRs from 22 cultivars were also investigated by RNA-seq to establish possible links between metabolite levels and the expression of candidate genes (Additional file 2: Table S1). Each sample was analyzed with three biological replicates. RNA-seq libraries were constructed by the Annoroad Gene Technology Corporation (Beijing, China) and sequenced on the Illumina Hiseq 4000 platform. RNA-seq data were processed and gene expression levels were calculated by fragments per kilobase per million mapped reads (FPKM) as previously described [15].

### Genotyping of candidate genes

The genomic regions of five candidate genes (including *MeMYB4*, *Me3GT*, *CYP79D1*, *CYP71E7b*, and *UGT85K5*) were amplified and the PCR products were sequenced by the Sanger method in 16~22 cassava accessions, of which half had high content of metabolites while another half had low content of the corresponding metabolites (Additional file 2: Table S8-12). The genomic DNA was extracted from selected cassava accessions using the Hi-DNAsecure Plant Kit (DP350, TIANGEN), and then applied as a template for PCR amplification with the following parameters: 94°C for 5 min, followed by 35 cycles of 94°C for 30 s, 55°C for 30 s, and 72°C for 90 s, and a final extension at 72°C for 10 min.

### Functional characterization of MeMYB4

The virus-induced gene silencing (VIGS) experiment was performed as previously described [10]. The partial coding sequence of *MeMYB4* was amplified from cassava cultivar SC205 and cloned into the pTRV2 vector with DNA sequencing verification. The recombinant plasmids *MeMYB4*-pTRV2 and pTRV1 were transformed into *Agrobacterium tumefaciens* GV3101 and thereafter syringe infiltrated into cassava leaves. After 14 days cultivation, the cassava leaves were collected for qRT-PCR as previously described [60]. The relative expression of *MeMYB4*, *MeCHI* (Sc07g012880), and *MeF3H* (Sc02g010090) was measured using the 2^-ΔΔCt^ method with three biological replicates (Additional file 2: Table S16).

Yeast one-hybrid (Y1H) assays were performed using the Matchmaker Gold Y1H Library Screening System according to the manufacturer’s instructions (Clontech, USA). Promoter fragments of *MeCHI* and *MeF3H* were amplified from SC205 by PCR (Additional file 2: Table S16), verified by DNA sequencing, and then ligated into the pHIS2.1 vector using the ClonExpress II one-step cloning kit (Vazyme, Nanjing, China). The recombinant pHIS2.1 vector was then transformed into yeast strain Y187 and yeast cells were plated onto selective medium (SD/-His/3-AT) to detect any self-activation. The pHIS2.1-MeCHI and pHIS2.1-MeF3H vectors were used as baits, while the full-length CDS of *MeMYB4* was fused at C-terminus of GAL4-AD in the pGADT7 vector as prey. The bait and prey vectors were then co-transformed into yeast strain Y187 by the PEG/LiAc method, and yeast cells were cultured on double dropout medium (SD/-Leu/-Trp) and triple dropout medium (SD/-Leu/-Trp/-His/3-AT) at 28°C.

### Dual luciferase assay of MeMYB4 and MeFLS1 promoters

To determine the effect of allele variations (at −661 bp upstream of *MeMYB4* and −446 bp upstream of *MeFLS1*) on the activity of *MeMYB4* and *MeFLS1* promoters, we performed a dual luciferase assay in tobacco (*N. benthamiana*). The promoter sequences (from 0 to −1000 bp) of *MeMYB4* (carrying C or T) and *MeFLS1* (carrying G or T) were cloned and inserted into the pGreenII0800-LUC vector. The recombinant constructs were transformed into tobacco leaves by injection as previously described [61]. The relative luciferase activity was examined using a dual-luciferase reporter assay system (E710, Promega, Madison, USA).

### In vitro enzyme assays of Me3GT and UGT85K4/UGT85K5

The full-length cDNAs of Me3GT (including allele1, allele2, A6T, G80D, S101C, T145A, S146G, P334S), UGT85K4, and UGT85K5 were cloned separately into the pGEX-6p-1 expression vector (Novagen), each with a glutathione S-transferase tag. Recombinant proteins were expressed in BL21 (DE3) cells (Novagen), and pelleted cells were re-suspended in lysis buffer (50 mM Tris–HCl with pH 8.0, 400 mM NaCl). The cells were disrupted using a high-pressure cracker and the supernatants of crude protein were obtained by centrifugation at 14,000*g* for 1 h. Glutathione Sepharose 4B agarose (GE Healthcare) was added to the supernatant containing the target proteins. After 1 h incubation, the mixture was transferred into a disposable column and then washed with lysis buffer (5 column volumes). The target proteins in collected fractions were verified by SDS–PAGE, and the purified recombinant proteins were used to perform enzyme assays.

The in vitro glycosyltransferase assay of Me3GT (allele1) was carried out in a final volume of 100 µl containing 200 µM flavonoid acceptor (containing kaempferol, quercetin, cyanidin, pelargonidin, apigenin, chrysin, naringenin, and eriodictyol, respectively), 1.5 mM UDP-sugar donor, 5 mM MgCl2, and 500 ng purified protein in Tris–HCl buffer (100 mM, pH 7.4), and the reaction mixture was incubated at 37 °C for 15 min. The *in vitro* glycosyltransferase assay of Me3GT (including allele1, allele2, A6T, G80D, S101C, T145A, S146G, P334S) was performed as above, but only using kaempferol as the acceptor. The generated contents of kaempferol 3-O-glucoside were used to evaluate the relative activity of Me3GT.

The *in vitro* glycosyltransferase assay of UGT85K4 and UGT85K5 was carried out in a final volume of 100 µl containing 200 µM acetone cyanohydrin, 1.5 mM UDP-sugar donor, 5 mM MgCl2, and 500 ng purified protein in Tris–HCl buffer (100 mM, pH 7.4), and the reaction mixture was incubated at 37 °C for 15 min. When the reaction was stopped, the mixture was filtered using a 0.2-mm Millipore filter and was then subjected to LC-MS analysis.

### Functional characterization of Me3GT and UGT85K5

The cassava common mosaic virus isolate CM (CsCMV-CM) based VIGS system [62] was used to verify the function of *Me3GT* and *UGT85K5*. The partial coding sequence was amplified from cassava cultivar SC205 and cloned into the CsCMV-CM vector with DNA sequencing verification. The recombinant plasmid was transformed into Agrobacterium tumefaciens GV3101 (psoup) and thereafter syringe infiltrated into cassava leaves. After 21 days cultivation, the cassava leaves were collected for qRT-PCR as previously described [60]. The relative expression of *Me3GT* and *UGT85K5* was measured using the 2^-ΔΔCt^ method with three biological replicates (Additional file 2: Table S16). The leaves of *Me3GT*-silenced and *UGT85K5*-silenced plants were used to examine the metabolite levels.

### Supplementary Information

**Additional file 1:** **Fig. S1.** Detected metabolites and their correlation coefficients. **Fig. S2.** Visualization of metabolite networks. **Fig. S3.** Functional annotation of genes responsible for variation in 5-O-p-coumaroylquinic acid content. **Fig. S4.** Functional annotation of genes responsible for variation in myricetin 3-O-galactoside content. **Fig. S5.** Functional annotation of genes responsible for variation in dihydrokaempferol content. **Fig. S6.** Functional validation of UGT85K4 and UGT85K5. **Fig. S7.** Expression correlation of CG metabolism genes based on 465 RNA-seq data. **Fig. S8.** Phylogeny of five representative wild accessions and 388 previously published cassava accessions.**Fig. S9.** Manhattan plot displaying the GWAS results of 16 flavones. **Fig. S10.** Manhattan plot displaying the GWAS results of lotaustralin. **Fig. S11.** Differences in CG related metabolites between five wild and 299 cultivated cassava accessions.

**Additional file 2:** **Table S1.** Summary of 299 cultivated and 19 wild accessions used in this work. **Table S2.** Detailed information of metabolites detected in the replicate 1 of this study. **Table S3.** Detailed information of metabolites detected in the replicate 2 of this study. **Table S4.** List of lead SNPs detected in mGWAS from two replications. **Table S5.** Summary of genome-wide significant associations identified in mGWAS. **Table S6.** Full lists of significant mGWAS associations. **Table S7. **Summary of 12 candidate genes assigned from mGWAS results. **Table S8. **Significant associated information of SNPs at the genomic region of Me3GT. **Table S9.** SNPs detected at the genomic region of CYP79D1. **Table S10.** SNPs detected at the genomic region of UGT85K5. **Table S11.** SNPs detected at the genomic region of CYP71E7b. **Table S12.** SNPs detected at the genomic region of MeMYB4. **Table S13.** Summary of 132 annotated metabolites involved in cassava domestication. **Table S14.** Summary of 14 flavones involved in SR development and co-located with SR weight per plant. **Table S15.** Expression levels of CG cluster genes in response to MeJA treatment and Xam inoculation. **Table S16.** The primers used in this study.

**Additional file 3.** Review history.

## Data Availability

The whole genome re-sequencing and RNA-Seq data have been deposited in the Sequence Read Archive (SRA) database under the NCBI BioProject accession PRJNA578024 [63]. Additional re-sequencing data of wild cassava accessions were obtained from the CassavaBase database [44] and the NCBI-SRA database under the accession number PRJNA234389 [64]. The metabolic profiling data generated in this study are included in Additional file 2: Table S2 and Table S3. The metabolic raw data are deposited at the EMBL-EBI MetaboLights database with the accession number MTBLS9027 [65]. No other scripts and software were used other than those mentioned in the “Methods” section.
