# Supplementary material for: Metabolic GWAS-based dissection of genetic basis underlying nutrient quality variation and domestication of cassava storage root
Source: Genome Biol. 2023 Dec 14;24:289. doi: 10.1186/s13059-023-03137-y (PMC10722858; doi:10.1186/s13059-023-03137-y)
Supplement: Supplementary file 1 — Additional file 1: Fig. S1. Detected metabolites and their correlation coefficients. Fig. S2. Visualization of metabolite networks. Fig. S3. Functional annotation of genes responsible for variation in 5-O-p-coumaroylquinic acid content. Fig. S4. Functional annotation of genes responsible for variation in myricetin 3-O-galactoside content. Fig. S5. Functional annotation of genes responsible for variation in dihydrokaempferol content. Fig. S6. Functional validation of UGT85K4 and UGT85K5. Fig. S7. Expression correlation of CG metabolism genes based on 465 RNA-seq data. Fig. S8. Phylogeny of five representative wild accessions and 388 previously published cassava accessions.Fig. S9. Manhattan plot displaying the GWAS results of 16 flavones. Fig. S10. Manhattan plot displaying the GWAS results of lotaustralin. Fig. S11. Differences in CG related metabolites between five wild and 299 cultivated cassava accessions. [file 13059_2023_3137_MOESM1_ESM.pdf]

Supplemental files

Metabolic GWAS-based dissection of genetic bases underlying nutrient quality variation and domestication of cassava storage root

Zehong Ding[1†], Lili Fu[1†], Bin Wang[2†], Jianqiu Ye[3], Wenjun Ou[3], Yan Yan[1,4], Meiying Li[1,4], Liwang Zeng[5,6], Xuekui Dong[7], Weiwei Tie[1,4,5], Xiaoxue Ye[1,4,5], Jinghao Yang[1,4], Zhengnan Xie[1,4], Yu Wang[1,4,5], Jianchun Guo[1,4], Songbi Chen[3], Xinhui Xiao[3], Zhongqing Wan[3], Feifei An[3], Jiaming Zhang[1,4], Ming Peng[1,4,5], Jie Luo[5,8*], Kaimian Li[3*], Wei Hu[1†*]

[*]**Correspondence**: Jie Luo (jie.luo@hainanu.edu.cn), Kaimian Li (likaimian@sohu.com), Wei Hu (huwei2010916@126.com).

[†]Zehong Ding, Wei Hu, Lili Fu, and Bin Wang contributed equally to this work.

E-mails:

Zehong Ding: dingzehong@itbb.org.cn; **Wei Hu**: huwei2010916@126.com; **Lili Fu**: fulili@itbb.org.cn; **Bin Wang**: wangbin@metware.cn; **Jianqiu Ye:** yejianqiu2006@126.com; **Wenjun Ou:** cassava6973@163.com; **Yan Yan**: yoyoyan7758@163.com; **Meiying Li**: limeiying@itbb.org.cn; **Liwang Zeng**: zengliwang@163.com; **Xuekui Dong**: dongxuekui.123@163.com; **Weiwei Tie**: tieweiwei@itbb.org.cn; **Xiaoxue Ye**: yexiaoxue@itbb.org.cn; **Jinghao Yang**: yangjinghao@itbb.org.cn; **Zhengnan Xie**: xiezhengnan@itbb.org.cn; **Yu Wang**: wangyu@itbb.org.cn; **Jianchun Guo:** guojianchun@itbb.org.cn; **Songbi Chen:** songbichen@hotmail.com; **Xinhui Xiao:** xiaoxinhui1983@163.com; **Zhongqing Wan:** wan_zhongqing@163.com; **Feifei An:** aff85110@163.com; **Jiaming Zhang**: zhangjiaming@itbb.org.cn; **Ming Peng:** pengming@itbb.org.cn; **Jie Luo**: jie.luo@hainanu.edu.cn; **Kaimian Li:** likaimian@sohu.com

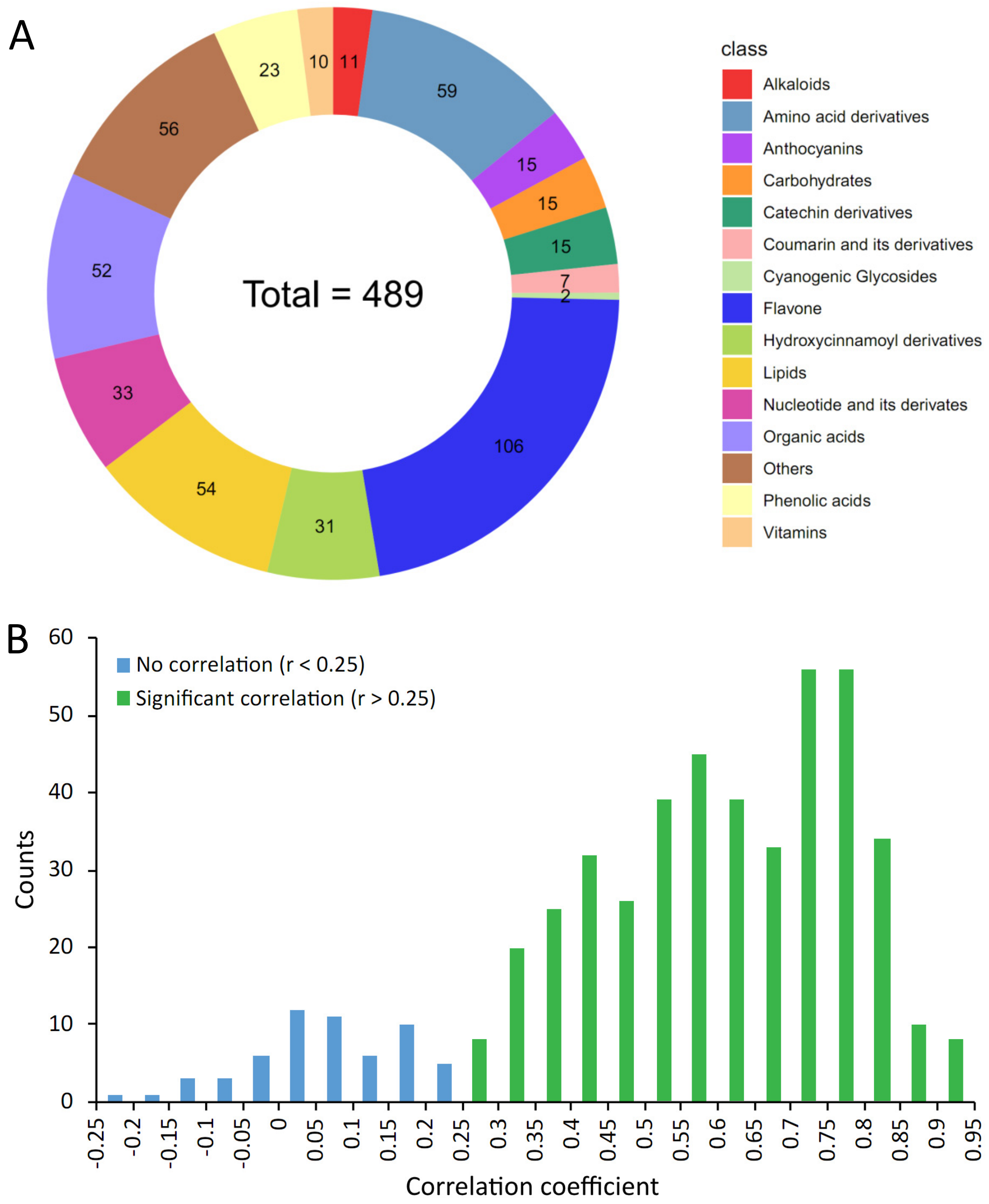

Fig. S1 Detected metabolites and their correlation coefficients.

(A) Number of annotated metabolites in each category.

(B) Correlation coefficients of annotated metabolites between two biological replicates.

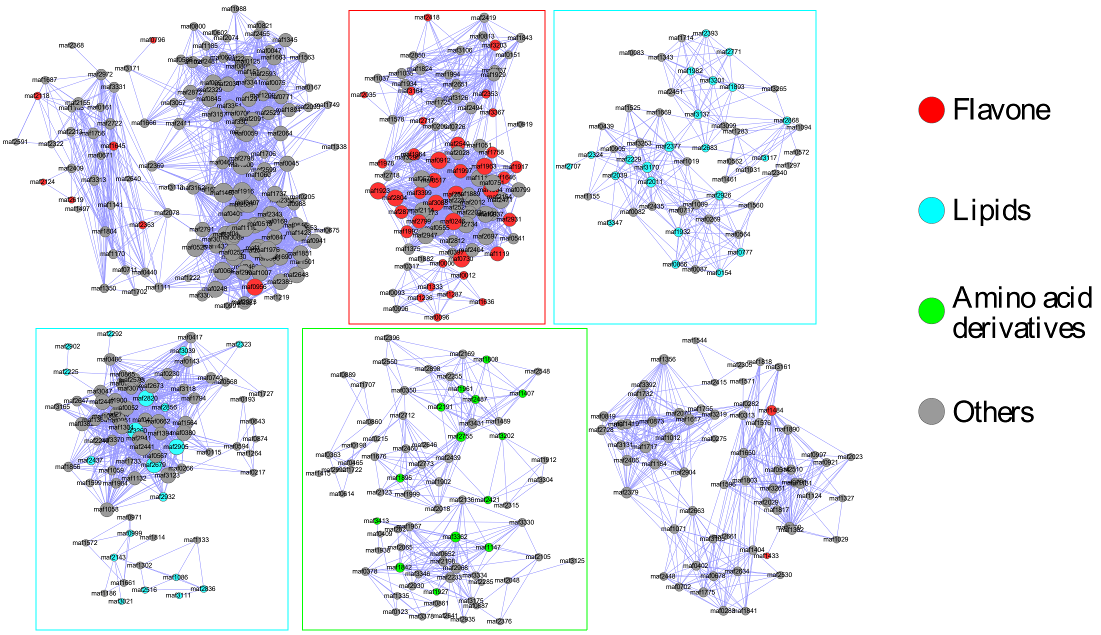

Fig. S2 Visualization of metabolite networks.

Nodes are metabolites while edges represent their correlation coefficients. The top six most abundant networks and the edges with Pearson's correlation coefficients greater than 0.8 are shown. Node size indicates its connectivity to others. Metabolites from distinct biochemical groups are indicated by different colors as shown on the right.

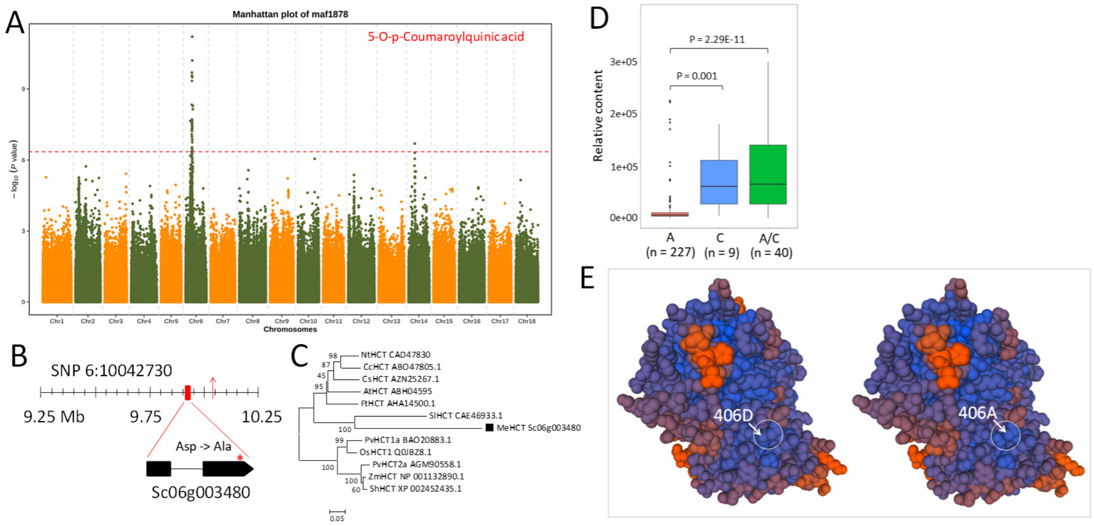

Fig. S3 Functional annotation of genes responsible for variation in 5-O-p-coumaroylquinic acid content.

(A) Manhattan plot displaying the GWAS results for 5-O-p-coumaroylquinic acid content.

(B) Gene model of Sc06g003480 (*MeHCT*). The lead SNP 6:10042730 is indicated by a vertical arrow. The causative SNP 6:9924983, which is located in exon 2 of *MeHCT*, is indicated by a red star.

(C) Phylogenetic tree of *MeHCT* and HCT genes from other species. Bootstrap values are indicated at each node.

(D) Boxplot displaying the relative content of 5-O-p-coumaroylquinic acid attributable to the possible causative SNP 6:9924983.

(E) Predicted 3D structure of MeHCT protein with the difference of D406A mutation.

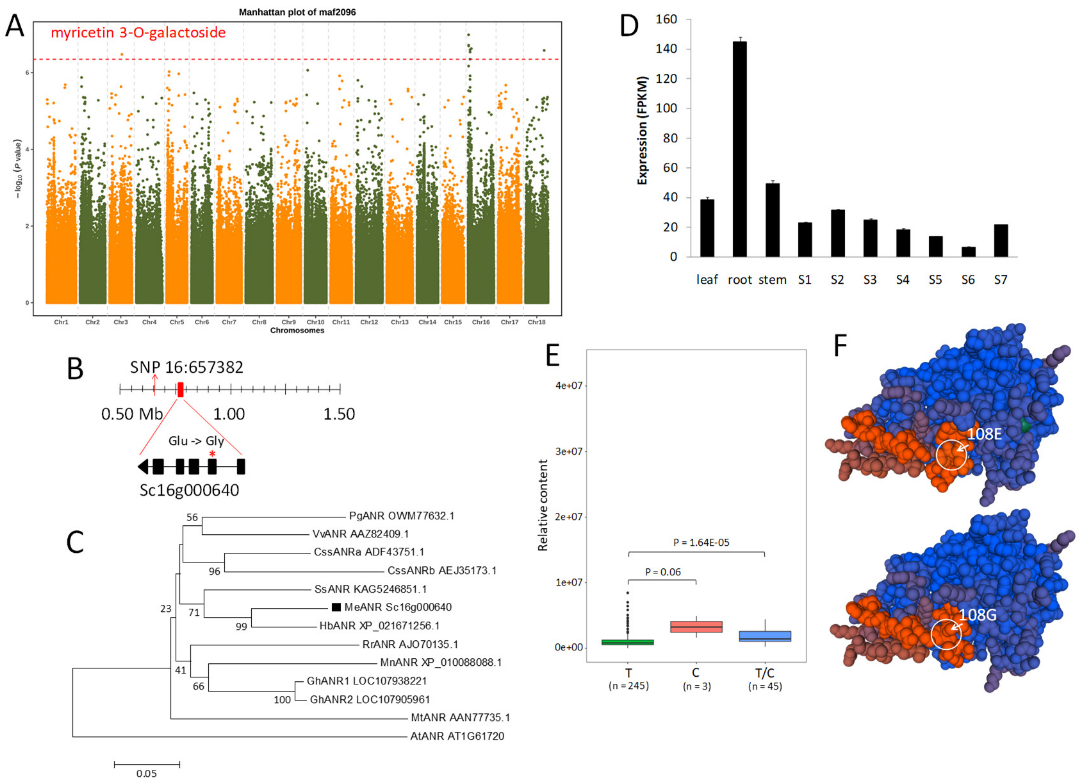

Fig. S4 Functional annotation of genes responsible for variation in myricetin 3-O-galactoside content.

(A) Manhattan plot displaying the GWAS results of myricetin 3-O-galactoside content.

(B) Gene model of Sc16g000640 (*MeANR*). The lead SNP 16:657382 is indicated by a vertical arrow. The causative SNP 16:760436, which located in the exon 2 of *MeANR*, is indicated by a red star.

(C) Phylogenetic tree of *MeANR* and ANR genes from other species. Bootstrap values are indicated at each node.

(D) Expression level of *MeANR* in leaf, root, stem, and among seven developmental stages (S1–S7) of storage root.

(E) Boxplot displaying the relative content of myricetin 3-O-galactoside based on the possible causative SNP 16:760436.

(F) Predicted 3D structure of MeANR protein with the difference of E108G mutation.

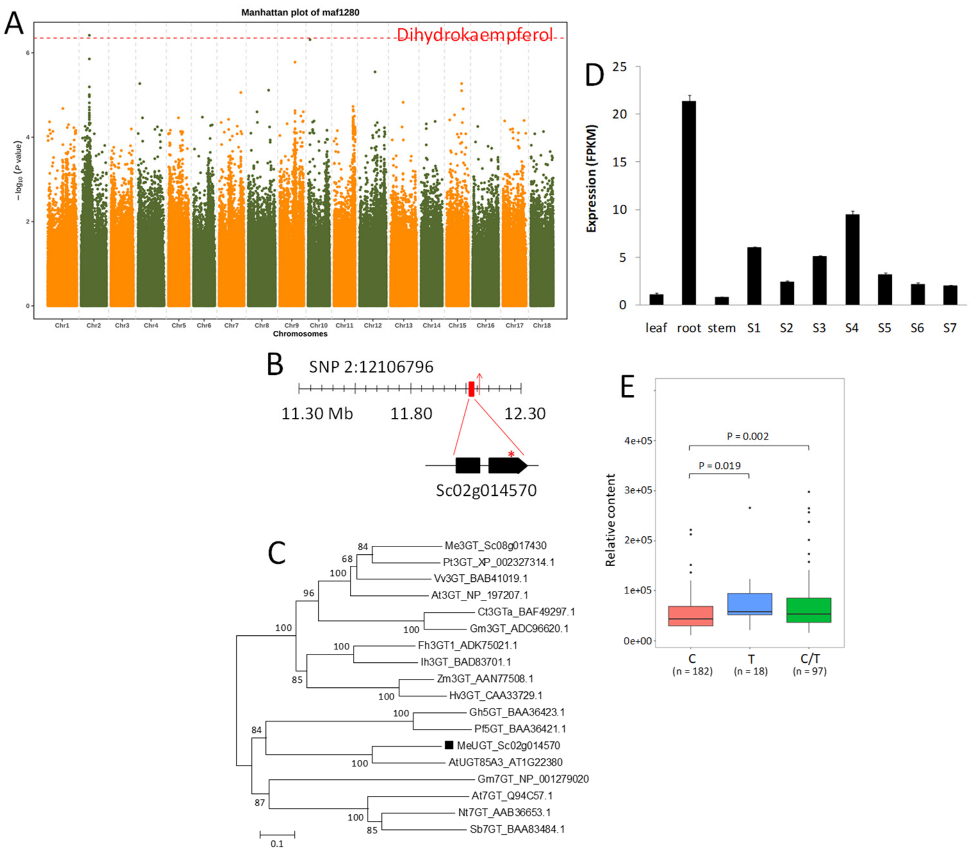

Fig. S5 Functional annotation of genes responsible for variation in dihydrokaempferol content.

(A) Manhattan plot displaying the GWAS results for dihydrokaempferol content.

(B) Gene model of Sc02g014570 (*MeUGT*). The lead SNP 2:12106796 is indicated by a vertical arrow. The possible causative SNP 2:12069387, which located in the exon 2 of *MeUGT*, is indicated by a red star.

(C) Phylogenetic tree of *MeUGT* and UGT genes from other species. Bootstrap values are indicated at each node.

(D) Expression level of *MeUGT* in leaf, root, stem, and among seven developmental stages (S1–S7) of storage root.

(E) Boxplot displaying the relative content of dihydrokaempferol based on the causative SNP 2:12069387.

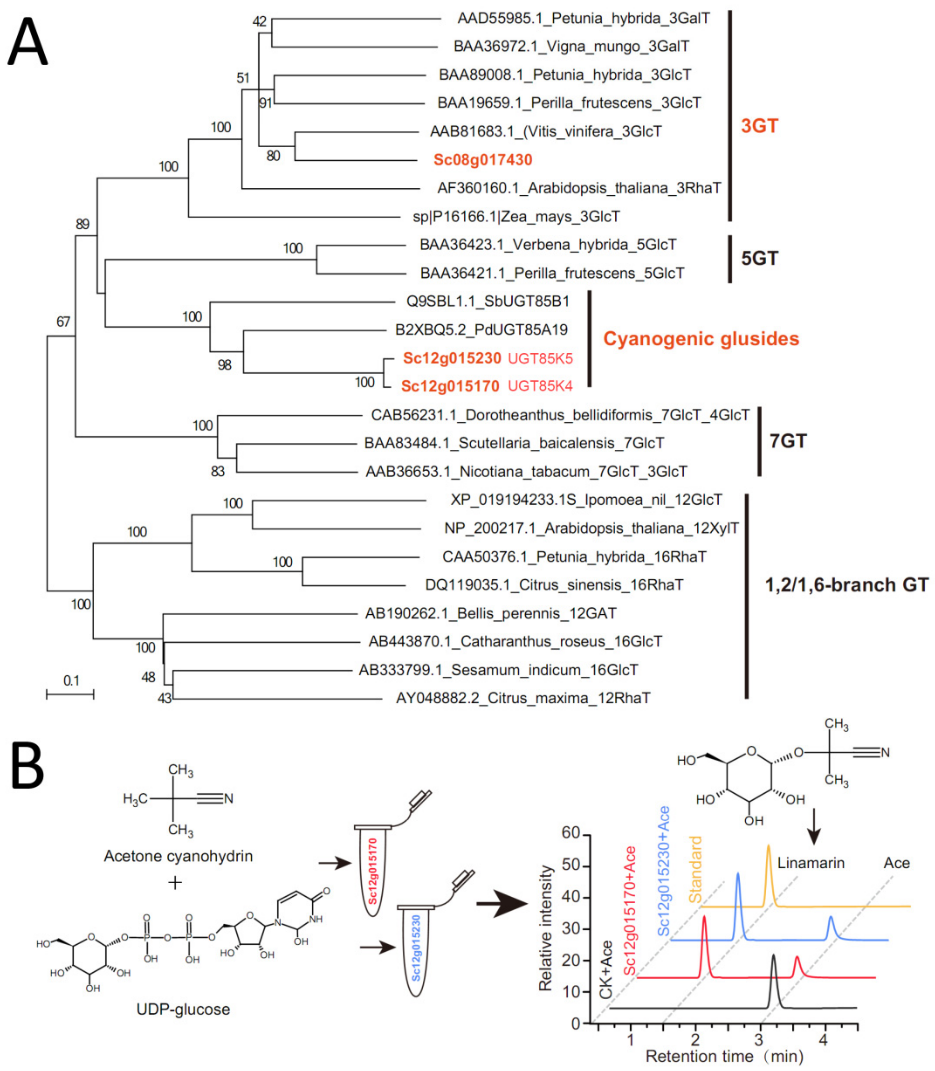

Fig. S6 Functional validation of UGT85K4 and UGT85K5.

(A) Phylogenetic tree of glucose transferases (GTs) from different plant species.

(B) HPLC chromatograms of the products of the reactions of UGT85K4 and UGT85K5, respectively, with UDP-glucose and acetone cyanohydrin.

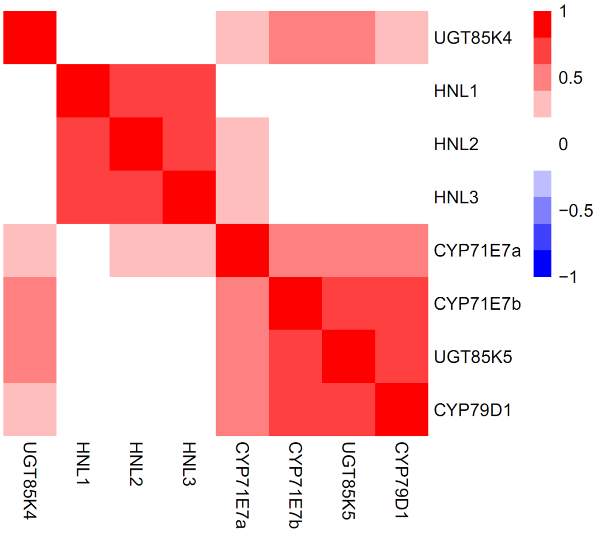

Fig. S7 Expression correlation of CG metabolism genes based on 465 RNA-seq data.

Note: These RNA-Seq data were derived from Fu et al. (2021) Large-scale RNAseq analysis reveals new insights into the key genes and regulatory networks of anthocyanin biosynthesis during development and stress in cassava. Ind Crop Prod, 169: 113627.

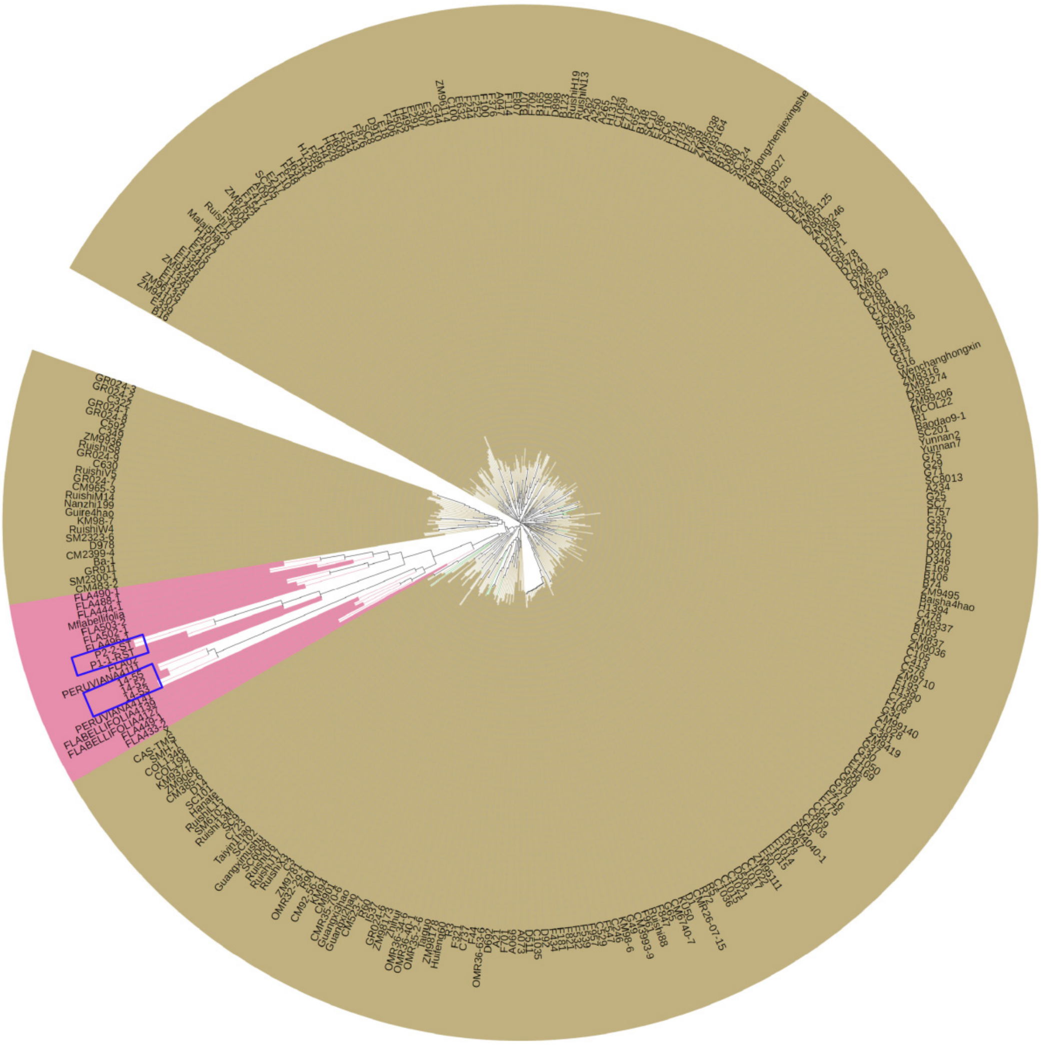

Fig. S8 Phylogeny of five representative wild accessions and 388 previously published cassava accessions.

The phylogeny of five representative wild accessions and 388 previously published (Hu et al., 2021) cassava accessions was generated using the neighbor-joining tree method with genome-wide SNPs. The wild and cultivated cassava accessions were highlighted by pink and brown, respectively. The five representative wild accessions used in this work were indicated with blue boxes.

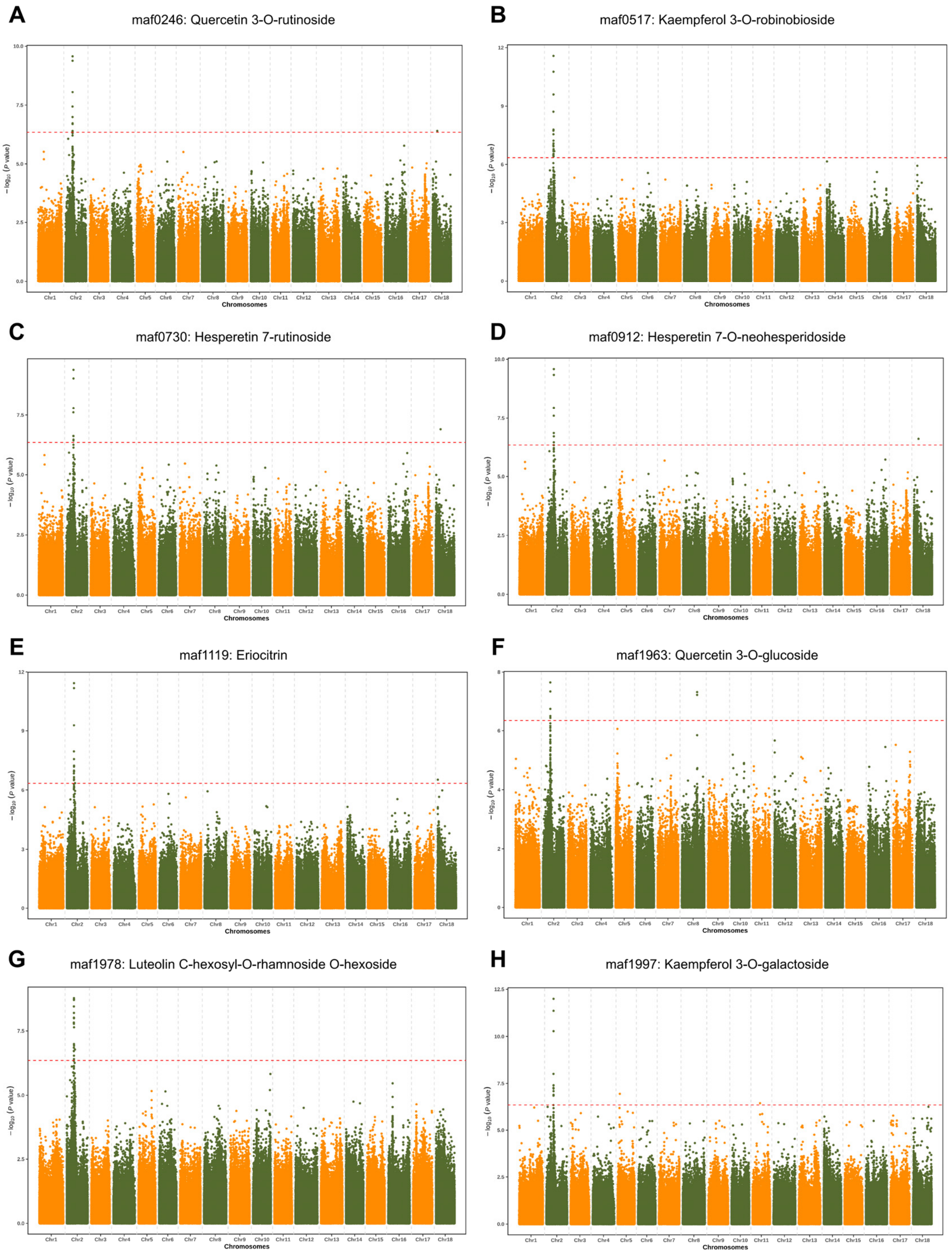

A
maf0246: Quercetin 3-O-rutinoside
B
maf0517: Kaempferol 3-O-robinobioside
C
maf0730: Hesperetin 7-rutinoside
D
maf0912: Hesperetin 7-O-neohesperidoside
E
maf1119: Eriocitrin
F
maf1963: Quercetin 3-O-glucoside
G
maf1978: Luteolin C-hexosyl-O-rhamnoside O-hexoside
H
maf1997: Kaempferol 3-O-galactoside
Chromosomes
$-\log_{10}$ (P value)
Chr1 Chr2 Chr3 Chr4 Chr5 Chr6 Chr7 Chr8 Chr9 Chr10 Chr11 Chr12 Chr13 Chr14 Chr15 Chr16 Chr17 Chr18

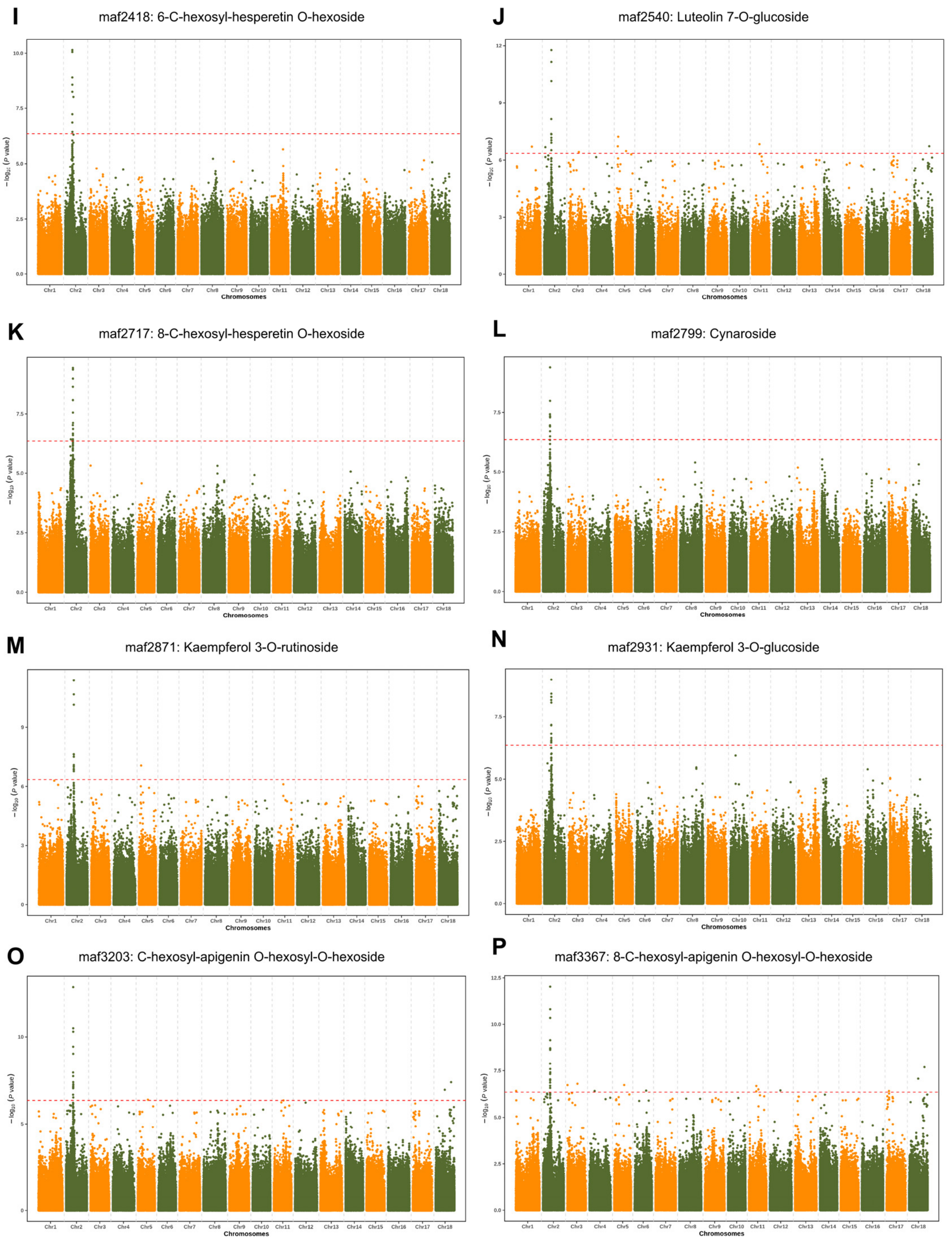

Fig. S9 Manhattan plot displaying the GWAS results of 16 flavones.

These flavones were co-located with the pGWAS signal for SR weight per plant on Chr2:12.84 Mb.

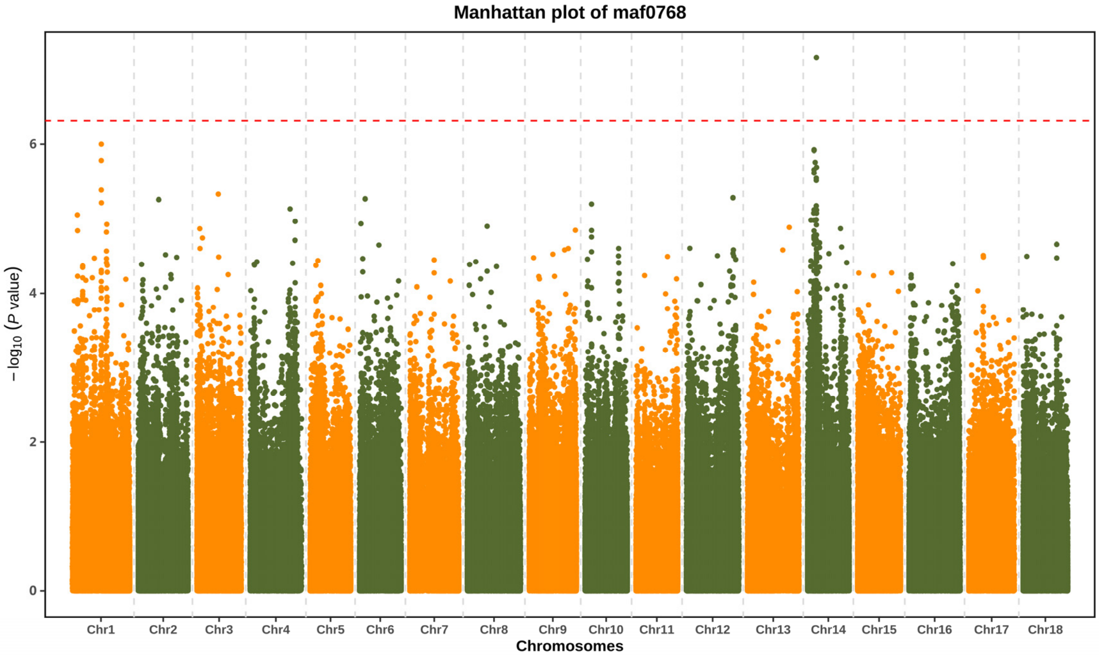

Fig. S10 Manhattan plot displaying the GWAS results of lotaustralin.

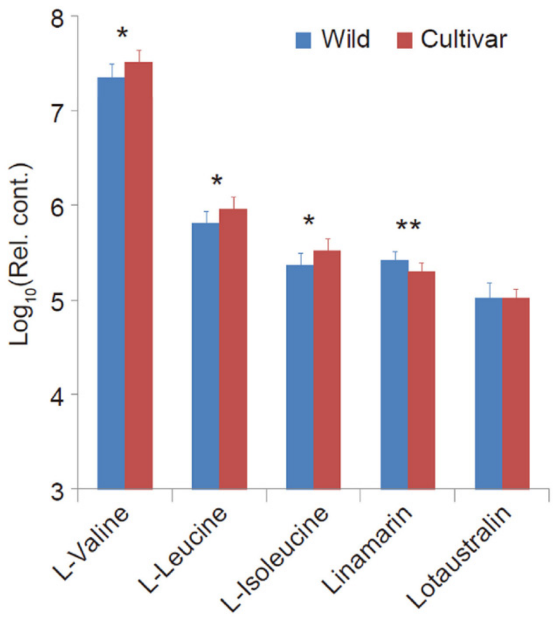

Fig. S11 Differences in CG related metabolites between wild (n = 5) and cultivated (n = 299) cassava accessions.

* and ** represent the significant differences at $P < 0.05$ and $P < 0.01$ based on T-tests, respectively.
