# Supplementary material for: Metabolic GWAS-based dissection of genetic basis underlying nutrient quality variation and domestication of cassava storage root
Source: Genome Biol. 2023 Dec 14;24:289. doi: 10.1186/s13059-023-03137-y (PMC10722858; doi:10.1186/s13059-023-03137-y)
Supplement: Supplementary file 3 — Additional file 3. Review history. [file 13059_2023_3137_MOESM3_ESM.docx]

Review History

**First round of review**

**Reviewer 1**

**Were you able to assess all statistics in the manuscript, including the appropriateness of statistical tests used?**

Yes, and I have assessed the statistics in my report.

**Comments to author:**

Authors conducted metabolic and phenotypic GWAS in 299 cultivated cassava accessions based on the previous resequencing data. Notably, they quantified 2,980 metabolites, of which 489 metabolites were annotated. So far, this appears to be the first application of mGWAS in a large number of accessions in cassava. The high number of SNPs and metabolites is essential, since it enables the authors to dissect the genetic basis of metabolites in storage root of cassava in high resolution. Notably, the authors identified 14 candidate genes responsible for the levels of metabolites. MeFLS1 was selected during cassava domestication to decrease quercetin decrease quercetin 3-O-glucoside content and increase storage root weight. The study appears well conducted and interesting; however, there are several points that need clarifications.

(1) The analysis for the decay of linkage disequilibrium (LD) in the cassava genome is essential in this study.

(2) Most of the causative SNPs are upstream, intergenic, downstream, intronic, UTR5, UTR3, synonymous. Therefore, it is necessary to sequence the 14 candidate genes in the GWAS population, and identify more useful SNPs to examine their effects on corresponding traits. This will be a strong supplemental evidence for the validation of the candidate genes.

(3) I suggest the authors to provide more detailed description of GWAS, PCA, and phylogenetic analysis. For example, it would be helpful if they demonstrate that the effects of population structure on GWAS was properly controlled.

(4) It is not clear how the candidate genes and causative SNPs were determined.

(5) L154-155, the claim is misleading, since the resequencing data is not generated in this study.

(6) Fig. S2 can be hardly read and has low resolution.

**Reviewer 2**

**Were you able to assess all statistics in the manuscript, including the appropriateness of statistical tests used?**

Yes, and I have assessed the statistics in my report.

**Comments to author:**

The manuscript by Ding and co-workers presents a comprehensive metabolic and phenotypic GWAS about the metabolites in storage root (SR) of cassava. The study offers useful resources for genetic improvement of the nutritional qualities in cassava. However, the serious problems are some loci mentioned in the manuscript which are not significant, and the selected genes are far away from the lead SNP. Additionally, the authors only used 5 wild accessions data to analyze the domestication event, how are the results reliable? It is necessary to consider whether the selected genes are appropriate, and provide more omics data to support the importance of the selected genes. Here, I have several major questions to this manuscript:

1, Line 157. The author mentioned two biological replicates per accession were collected for metabolite profiling, why did not test three biological replicates per accession? However, there are only one set of metabolite profiling data was shown in the supplementary table. Please list the detailed data of these two biological replicates, and analyze the repeatability of these two biological replicates. In addition, in order to confirm the reliability of metabolome and GWAS more rigorously, please add the GWAS results of each biological replicates of the metabolite profiling.

2, The metabolites were measured from single year source, and is likely that their content is influenced by the environment. How than could be heritability calculated?

3, The lead SNP 1:29336904 is 138kb away from the possible causative SNP 1:29198131. Whether this causative SNP is associated with the lead SNP? And if the p value of the causative SNP exceeded the threshold? Similar confusions were also found in Figure 2, Figure 3, Figure 4, Figure 5, Figure S4, S5 and Table S4. Since the genes mentioned in the manuscript are far away from the lead SNP, whether these genes play key roles in controlling metabolite contents need to be further considered.

4, Fig. 2D. The relationship between 8-C-hexosyl-kaempferol O-hexoside, kaempferol 3-O-glucoside, quercetin 3-O-glucoside, pelargonidin-3-O-glucoside and cyanidin-3-O-glucoside needs to be explained.

5, What is the structure of 8-C-hexosyl-kaempferol O-hexoside? Is it 3-O glycoside? Could Me3GT catalyze its synthesis directly? Furthermore, the function of Me3GT should be validated in vivo. That should be done in other biosynthesis genes.

6, Fig. 3B only shows the GWAS of linamarin. The logP of SNP Chr16:32817362 didn't attach to the significant threshold. How about lotaustralin? The synthesis of lotaustralin and linamarin are catalyzed by CYP79D1, CYP71E7 and UGT85K4/UGT85K5 according to Fig. 3A, but why lotaustralin and linamarin are not localized to the same hot spots?

7, Many reports showed that CG accumulation is induced by jasmonic acid (JA), how is the relationship between JA and CG biosynthesis? Are the cluster genes expression induced by JA? As the CG contents also are related with resistance to biotic stresses, are there any different resistances or phenotypes for different CG contents haplotypes?

8, Line 230, the authors observed Sc16g010850 was subsequently assigned as a candidate gene for linamarin based on the marginal significant correlation (Cor = 0.41, P = 0.06) between the expression of this gene and linamarin content. P-value called into question the reliability of the result. I didn't find the detail about the correlation analysis between the expression of this gene and linamarin content. And I didn't find the qPCR primers in the Supplement tables. Was the expression of Sc16g010850 in the analysis from RNA-Seq? Please add detailed data.

9, In Fig. 3E-H, why the linamarin content is not significantly different between Hap.GAA and Hap.TGT?

10, Line 379 "linamarin is more abundant (~95% of CG) than lotaustralin in cassava SR". I noticed that this was not intention of G. Padmaja and Dr. Keith H. Steinkraus (1995). Please check it and give other more evidence for the predominance of linamarin in cassava HCN.

11, It is necessary to verify the function of these non-synonymous mutations on gene function, and the function of mutations in promoters on the gene expression level.

12, Fig. 4C, the phenotypic data described in the figure needs to be shown in the supplementary tables.

13, Figure 4. For the function of MeMYB4, it is really very strange. Because MYB4 belongs to the subgroup 4 of MYB TFs, which are the repressors on phenylalanine metabolism, how can you explain your results conflict with previous reports? The author mentioned MeMYB4 could positively control cyanidin 3-O-glucoside and delphinidin 3-O-rutinoside content via interaction with the promoter of MeCHI and MeF3H. However, the expression level of MeCHI and MeF3H in MeMYB4 transient silencing lines should be added to further prove this suggestion.

14, There are only average metabolite contents in wild cassava accession. The detailed metabolite profiling of 5 wild cassava accessions should be listed respectively.

15, For the figure 5 about the domestication story, it is completely not reliable due to only 5 wild accessions analyzed.

16, To provide insights into the metabolic changes that have taken place during cassava domestication, the authors compared metabolite profiles between 299 cultivated and five representative wild cassava accessions, and identified 132 annotated differentially accumulated metabolites (DAMs), setting the variable importance in projection (VIP) ≥1 and |fold-change| ≥1.5. However, I noted that many metabolites in the VIP didn't meet this criterion in Table S5.

17, Line 303, base on the mGWAS and pGWAS co-locations, the authors revealed that mGWAS signals for 16 flavones were co-located with the pGWAS signal for SR weight per plant on Chr2:12.84 Mb. Please point out these 16 flavones and show the mGWAS of them in figure.

18, Line 312, "The expression of MeFLS1 coincided with the changes in quercetin 3-O-glucoside contents among different tissues". The expression of MeFLS1 was extremely low in S1-S7, while quercetin 3-O-glucoside accumulated in SR according to Fig. 5L and 5M, which didn't support the conclusion. Moreover, the data presented in Fig. 5M were not consistent with Table S6.

19, In Fig. 5O, P=0.08 cannot indicate a significant difference between them. The method of significance test should be described in figure legends.

20, Improper citation, eg, Line 335.

21, There is description of the principal component analysis in the section Methods, but is not relevant results in the manuscript.

**Authors Response**

**Point-by-point responses to the reviewers’ comments:**

Reviewer #1:

Authors conducted metabolic and phenotypic GWAS in 299 cultivated cassava accessions based on the previous resequencing data. Notably, they quantified 2,980 metabolites, of which 489 metabolites were annotated. So far, this appears to be the first application of mGWAS in a large number of accessions in cassava. The high number of SNPs and metabolites is essential, since it enables the authors to dissect the genetic basis of metabolites in storage root of cassava in high resolution. Notably, the authors identified 14 candidate genes responsible for the levels of metabolites. MeFLS1 was selected during cassava domestication to decrease quercetin decrease quercetin 3-O-glucoside content and increase storage root weight. The study appears well conducted and interesting; however, there are several points that need clarifications.

*Answer: Thank you very much for your comments. Your insights and suggestions are extremely important and reasonable, which helped usto improve the quality of the manuscript and avoid errors in our interpretation. Based on your valuable suggestions, we have made careful revisions (see the details below) on our manuscript. We hope that these revisions are satisfactory.*

(1) The analysis for the decay of linkage disequilibrium (LD) in the cassava genome is essential in this study.

*Answer: Thank you very much for your valuable comments. We performed LD decay analysis using the 299 cassava samples and found that the LD extent was ~205 kb (r2 = 0.1, Reply Fig. 1). This parameter was used later for the identification of candidate genes in mGWAS analysis.*

(2) Most of the causative SNPs are upstream, intergenic, downstream, intronic, UTR5, UTR3, synonymous. Therefore, it is necessary to sequence the 14 candidate genes in the GWAS population, and identify more useful SNPs to examine their effects on corresponding traits. This will be a strong supplemental evidence for the validation of the candidate genes.

*Answer: Thank you very much for your valuable comments. We agree with your opinion. We re‐sequenced the genomic regions of five crucial candidate genes presented in the main figure (including MeMYB4, Me3GT, CYP79D1, CYP71E7b, and UGT85K5) in 16~22 cassava accessions, of which half had high content of metabolites while another half had low content of the corresponding metabolites (Table. S8‐S12).*

*Totally eight SNPs were identified in the genomic region of MeMYB4 (Table. S12). SNP1 showed highly significant associations with cyanidin 3‐O‐glucoside, delphinidin 3‐O‐rutinoside, and SR endothelium color, and it is the causative SNP (Chr2:6158256) presented in our manuscript. These results confirmed the accuracy of the causative SNP Chr2:6158256 (SNP1). Moreover, our dual luciferase assay further demonstrated that SNP Chr2:6158256 (SNP1) affects the activity of MeMYB4 promoter (Fig. 4H), therefore we keep it as the causative SNP.*

*In total, 13 SNPs (including two synonymous, six nonsynonymous, and five at the promoter area) were identified in the genomic region of Me3GT, and they showed highly significant associations with the levels of 8‐C‐hexosyl‐kaempferol O‐hexoside (Table. S8). The five SNPsin the promoter are not likely the causative SNPs as the expression of Me3GT showed no significant (P = 0.13) correlation with the content of 8‐C‐hexosyl‐kaempferol O‐hexoside across 22 cassava cultivars. Of the six nonsynonymous mutations, five are not detected in the mGWAS population via re‐ sequencing, while the remaining one (SNP13) is the causative SNP (Chr8:38681061) presented in our manuscript.*

*Highly significant associations were identified between three SNPs (SNP1‐SNP3) in the promoter of CYP79D1 and the levels of linamarin (Table. S9). However, they are not likely the causative SNPs since no significant (P = 0.84) correlation was observed between the expression of CYP79D1 and the levels of linamarin across 22 cassava cultivars.*

*In total, 13 SNPs were identified in the genomic region of CYP71E7b. However, none of them showed highly significant associations with the levels of linamarin (Table. S11).*

*Totally 16 SNPs were identified in the genomic region of UGT85K5. Of which, three SNPs (SNP14‐ SNP16) in the 3’UTR region showed highly significant associations with the levels of linamarin (Table. S10). SNP16 is the causative SNP (Chr12:35953769) presented in our manuscript, while SNP14‐SNP15 are newly identified by PCR‐based sequencing but not detected in the mGWAS population by re‐sequencing, therefore we still keep SNP16 (Chr12:35953769) asthe causative SNP. Taken together, more SNPs were detected in the genomic region of five candidate genes by PCR‐ based sequencing. These information is helpful for the validation of causative SNPs as well as candidate genes. These contents were revised in the manuscript accordingly (Lines 241‐245, Lines 281‐291, and Lines 305‐309).*

(3) I suggest the authors to provide more detailed description of GWAS, PCA, and phylogenetic analysis. For example, it would be helpful if they demonstrate that the effects of population structure on GWAS was properly controlled.

*Answer: Thank you very much for your valuable comments. We have provided more detailed description of GWAS, PCA, and phylogenetic analysis in the revised manuscript.*

*(a) For the GWAS analysis, population structure was calculated by the ADMIXTURE software, kinship was calculated by the TASSEL program, and they were incorporated in a fixed and random effect MLM to control false positives (Lines 607‐610).*

*(b) For the phylogenetic analysis, the metabolic data generated from 299 cultivated cassava accessions were log2‐transformed to construct a neighbor‐joining tree for demonstration of the cassava population structure. The neighbor‐joining tree was constructed based on the pairwise population distance by the software PHYLIP v3.69 and then visualized by the software MEGA6 (Lines 652‐656).*

*(c) For the PCA analysis, this part was removed from the Methods section since there were not relevant results in the manuscript. These contents were revised accordingly in the manuscript.*

(4) It is not clear how the candidate genes and causative SNPs were determined.

*Answer: Thank you very much for your valuable comments. The related contents were added in the “Mining of candidate genes and causative SNPs” section (Lines 625‐635) as follows:*

*Mining of candidate genes and causative SNPs Combined biological and bioinformatics approaches (Chen et al. 2014) were used to mine candidate genes by (1) looking for a protein or protein cluster that is biochemically relevant to the associated metabolites encoded at these loci; (2) performing cluster analysis of candidate genes relative to homologous genes with known function; and (3) cross‐referencing with statistical results of Pearson correlation between the metabolic levels and candidate gene expression in different tissues or cultivars. The SNPs located within, 2 kb upstream, or 2 kb downstream of the candidate genes were used to examine their associations with the corresponding metabolic traits, and the significant SNPs were considered as possible causative SNPs underlying metabolites.*

*References: Chen W, Gao Y, Xie W, Gong L, Lu K, Wang W, et al. Genome‐wide association analyses provide genetic and biochemical insights into natural variation in rice metabolism. Nat Genet. 2014, 46(7): 714‐721.*

(5) L154-155, the claim is misleading, since the resequencing data is not generated in this study.

*Answer: Thank you very much for your valuable comments. We agree with your opinion and revised this sentence as: “The whole genome re‐sequencing of 299 cassava cultivars was performed by our previously study (Hu et al. 2021), yielding a total of 1,155,988 high‐ confidence SNPs that were subsequently used for mGWAS analysis (Lines 157‐159)”.*

*References: Hu W, Ji C, Liang Z, Ye J, Ou W, Ding Z, et al. Resequencing of 388 cassava accessions identifies valuable loci and selection for variation in heterozygosity. Genome Biol. 2021;22(1):316.*

(6) Fig. S2 can be hardly read and has low resolution.

*Answer: Thank you very much for your valuable suggestions. Fig. S2 has been revised and its resolution is greatly improved now.*

Reviewer #2:

The manuscript by Ding and co-workers presents a comprehensive metabolic and phenotypic GWAS about the metabolites in storage root (SR) of cassava. The study offers useful resources for genetic improvement of the nutritional qualities in cassava. However, the serious problems are some loci mentioned in the manuscript which are not significant, and the selected genes are far away from the lead SNP. Additionally, the authors only used 5 wild accessions data to analyze the domestication event, how are the results reliable? It is necessary to consider whether the selected genes are appropriate, and provide more omics data to support the importance of the selected genes. Here, I have several major questions to this manuscript:

*Answer: Thank you very much for your comments. Your insights and suggestions are extremely important and reasonable, which helped usto improve the quality of the manuscript and avoid errors in our interpretation. Based on your valuable suggestions, we have made careful revisions (see the details below) on our manuscript. We hope that these revisions are satisfactory.*

1, Line 157. The author mentioned two biological replicates per accession were collected for metabolite profiling, why did not test three biological replicates per accession? However, there are only one set of metabolite profiling data was shown in the supplementary table. Please list the detailed data of these two biological replicates, and analyze the repeatability of these two biological replicates. In addition, in order to confirm the reliability of metabolome and GWAS more rigorously, please add the GWAS results of each biological replicates of the metabolite profiling.

*Answer: Thank you very much for your valuable comments.*

*We agree with your opinion that three biological replicates are better than two biological replicates per accession. We did two biological replicates in this work due to the limited funding. We hope that you can understand our trouble. The mGWAS results are independently verified through other molecular experiments (e.g., in vitro enzyme assays and in vivo gene silencing analyses) to ensure their reliability. According to your suggestion, we listed the detailed data of these two biological replicates in Table. S2 and Table. S3, and analyzed their repeatability. Among the 489 annotated metabolites, 431 showed significant (r > 0.25 and P < 0.01) correlations between the two biological replicates (Additional file 1: Fig. S1B), suggesting a stable repeatability (Lines 137‐ 139).*

*We also listed the mGWAS results of each biological replicates in Table. S4. We identified 10,666 and 9,848 lead SNPs corresponding to 1515 and 1576 metabolites in replicate 1 and replicate 2 (Table. S5), respectively, with 2263 lead SNPs and 334 metabolites determined both in these two replicates. These results suggest a good reliability of mGWAS (Lines 159‐162).*

2, The metabolites were measured from single year source, and is likely that their content is influenced by the environment. How than could be heritability calculated?

*Answer: Thank you very much for your valuable comments. As described in Bradbury et al. (2007), broad‐sense heritability (H2 ) was calculated by using the following formula: H2 = var(G)/var(G)+var(E), in which var(G) and var(E) are the genetic and environmental variances, respectively. In ourstudy, var(E) isthe residual variance from the cMLM model. These contents were revised in the manuscript accordingly (Lines 610‐612).*

3, The lead SNP 1:29336904 is 138kb away from the possible causative SNP 1:29198131. Whether this causative SNP is associated with the lead SNP? And if the p value of the causative SNP exceeded the threshold? Similar confusions were also found in Figure 2, Figure 3, Figure 4, Figure 5, Figure S4, S5 and Table S4. Since the genes mentioned in the manuscript are far away from the lead SNP, whether these genes play key roles in controlling metabolite contents need to be further considered.

*Answer: Thank you very much for your valuable comments. We agree with your opinion. As described in the “Mining of candidate genes and causative SNPs” section, candidate genes were mined by combined biological and bioinformatics approaches: (1) looking for a protein or protein cluster that is biochemically relevant to the associated metabolites encoded at these loci; (2) performing cluster analysis of candidate genes relative to homologous genes with known function; and (3) cross‐referencing with statistical results of Pearson correlation between the metabolic levels and candidate gene expression in different tissues or cultivars. The distance between the candidate genes and lead SNPs is set to 200 kb, according to the LD decay analysis (LD = 205 kb, r 2 = 0.1; see the details in question Q1 of review 1). The SNPs located within, 2 kb upstream, or 2 kb downstream of the candidate genes were used to examine their associations with the corresponding metabolic traits, and the significant SNPs were considered as possible causative SNPs underlying metabolites. That’s an explanation why some causative SNPs are far away from the lead SNPs.*

*We checked the associations between causative SNPs and lead SNPs, respectively, based on the LD heatmap (Reply Fig. 2). Most associations between the causative SNPs and lead SNPs are OK, except low LD was observed for the region of CG biosynthesis genes. The association looks weird between the causative SNP and lead SNP for two candidate genes (MeGBSS2 and MeGT1), therefore those related contents were removed in the revised manuscript. The p‐ value of causative SNPs was calculated by one‐way ANOVA on the corresponding metabolite. We regard the statistic is significant if the p‐value exceeds the threshold which is determined by 0.05/N (N is the total number of SNPs in the candidate gene region).*

*To further verify the roles of causative SNPs and candidate genes,*

*(1) We performed a dual luciferase assay to determine the effect of causative SNPs (at −661 bp upstream of MeMYB4) on the activity of MeMYB4 promoters. The promoter regions of MeMYB4 carrying C had higher activities than those carrying T (Fig. 4H), supporting that the causative SNPs affect the expression of MeMYB4.*

*(2) We selected three genes (including Me3GT, UGT85K4, and UGT85K5) to perform virus‐ induced gene silencing (VIGS) experiments for their functional validation. However, this job has significantly been delayed due to the serious influence of COVID‐19 in our province. We believe that this concern can be resolved in the next round of review because the deadline of this round is coming. \*

4, Fig. 2D. The relationship between 8-C-hexosyl-kaempferol O-hexoside, kaempferol 3-O-glucoside, quercetin 3-O-glucoside, pelargonidin-3-O-glucoside and cyanidin-3-O-glucoside needs to be explained.

*Answer: Thank you very much for your valuable comments. The common characteristic of 8‐ C‐hexosyl‐kaempferol O‐hexoside, kaempferol 3‐O‐glucoside, quercetin 3‐O‐glucoside, pelargonidin‐3‐O‐glucoside, and cyanidin‐3‐O‐glucoside is that they all contain a hexoside. Here we used kaempferol, quercetin, pelargonidin, and cyanidin (instead of 8‐C‐hexosyl‐ kaempferol because of its scarcity) as acceptors to validate that Me3GT can add a hexose to the flavonoid mother nucleus, producing kaempferol 3‐O‐glucoside, quercetin 3‐O‐glucoside, pelargonidin‐3‐O‐glucoside, and cyanidin‐3‐O‐glucoside, respectively.*

5, What is the structure of 8-C-hexosyl-kaempferol O-hexoside? Is it 3-O glycoside? Could Me3GT catalyze its synthesis directly? Furthermore, the function of Me3GT should be validated in vivo. That should be done in other biosynthesis genes.

*Answer: Thank you very much for your valuable comments. 8‐C‐hexosyl‐kaempferol O‐ hexoside is similar to the structure of kaempferol 3‐O‐glucoside, as they both contain a O‐ linked hexose modification on kaempferol. Our in vitro enzymatic activity assay confirmed that Me3GT has 3‐O‐glucosyltransferase activity (e.g., catalyze kaempferol to generate kaempferol 3‐O‐glucoside) but do not have 5‐O‐glucosyltransferase or 7‐O‐glucosyltransferase activities, therefore we believe that 8‐C‐hexosyl‐kaempferol O‐hexoside belongs to 3‐O glycoside. However, due to the scarcity of 8‐C‐hexosyl‐kaempferol, we are difficult to verify whether Me3GT could directly catalyze the synthesis of 8‐C‐hexosyl‐kaempferol O‐hexoside.*

*As suggested, we performed virus‐induced gene silencing (VIGS) experiments to validate the function of Me3GT and other candidate genes in vivo. However, this job has significantly been delayed due to the seriousinfluence of COVID‐19 in our province. We believe that this concern can be resolved in the next round of review.*

6, Fig. 3B only shows the GWAS of linamarin. The logP of SNP Chr16:32817362 didn't attach to the significant threshold. How about lotaustralin? The synthesis of lotaustralin and linamarin are catalyzed by CYP79D1, CYP71E7 and UGT85K4/UGT85K5 according to Fig. 3A, but why lotaustralin and linamarin are not localized to the same hot spots?

*Answer: Thank you very much for your valuable comments. The P‐value of SNP Chr16:32817362 is 1.95e‐07, which means the corresponding ‐log10P equals to 6.71. It is actually significant because the significant threshold of mGWAS wassetto P ≤ 4.43e‐07 (‐log10P = 6.35) after Bonferroni correction. There was only one significant mGWAS association for lotaustralin, and its manhattan plot was added in Fig. S9.*

*Despite the same set of enzymes (including CYP79D1, CYP71E7 and UGT85K4/UGT85K5) are responsible for the biosynthesis of linamarin and lotaustralin, they are produced from L‐valine and L‐isoleucine, respectively. Therefore, L‐valine and L‐isoleucine are, at least, key factors influencing the biosynthesis of linamarin and lotaustralin, leading to different levels of these two compounds. In cassava SR, linamarin is more abundant (~95% of CG) than lotaustralin. Moreover, there is only a moderate Pearson correlation (R = 0.74) between the levels of linamarin and lotaustralin in cassava SR. These factors may cause the location of linamarin and lotaustralin at different hot spots.*

7, Many reports showed that CG accumulation is induced by jasmonic acid (JA), how is the relationship between JA and CG biosynthesis? Are the cluster genes expression induced by JA? As the CG contents also are related with resistance to biotic stresses, are there any different resistances or phenotypes for different CG contents haplotypes?

*Answer: Thank you very much for your valuable comments.*

*We harvested storage roots of cassava SC8,sliced them into pieces with ~3 mm thick, and then treated with 5 mM MeJA solution. RNA‐seq results revealed that UGT85K4, CYP71E7b, and CYP79D1 were significantly induced at 6 h or 12 h after MeJA treatment (Reply Table. S1).*

*Similarly, CYP71E7b and CYP79D1 were significantly induced by Xam infection at 7 dpi, although UGT85K4 was depressed (Reply Table. S2). We also examined the relationship between CBB resistance (Xam infection) and the haplotypes of CG biosynthesis genes (CYP79D1, CYP71E7b, and UGT85K5) in 96 cassava accessions, and found that they are not significantly (p‐value = 0.66) relevant.*

*These results indicate that CG biosynthesis is related to MeJA treatment and biotic stresses, but the resistance to biotic stresses (e.g., CBB resistance) cannot be simply determined by the haplotypes of CG biosynthesis genes. These results were discussed in Lines 465‐474 in the manuscript. Hope your concerns have been satisfactorily resolved.*

8, Line 230, the authors observed Sc16g010850 was subsequently assigned as a candidate gene for linamarin based on the marginal significant correlation (Cor = 0.41, P = 0.06) between the expression of this gene and linamarin content. P-value called into question the reliability of the result. I didn't find the detail about the correlation analysis between the expression of this gene and linamarin content. And I didn't find the qPCR primers in the Supplement tables. Was the expression of Sc16g010850 in the analysis from RNA-Seq? Please add detailed data.

*Answer: Thank you very much for your valuable comments. The expression of Sc16g010850 was analyzed by RNA‐seq with three biological replicates. The Pearson correlation between the expression of Sc16g010850 and the relative content of linamarin was calculated in SRs from 22 cultivars (Reply Fig. 3). However, the related contents were excluded from our manuscript because of the weird LD between the causative SNP and lead SNP of this gene.*

9, In Fig. 3E-H, why the linamarin content is not significantly different between Hap.GAA and Hap.TGT?

*Answer: Thank you very much for your valuable comments.*

*We checked the data and found that the difference of linamarin content between Hap.GAA and Hap.TGT is significant (P = 0.044) based on the ANOVA test, but not significant (P < 0.07) based on the Duncan’s multiple range test. This might be caused by the marginally significantly (P = 0.07) difference between A‐allele and T‐allele of UGT85K5 (Fig. 3G).*

10, Line 379 "linamarin is more abundant (~95% of CG) than lotaustralin in cassava SR". I noticed that this was not intention of G. Padmaja and Dr. Keith H. Steinkraus (1995). Please check it and give other more evidence for the predominance of linamarin in cassava HCN.

*Answer: Thank you very much for your valuable comments. We updated the references (Nartey 1968; Bisset et al. 1969) for the predominance of linamarin in cassava HCN (Line 445).*

*References: Nartey F. Studies on cassava, Manihot utilissima Pohl—I. Cyanogenesis: the biosynthesis of linamarin and lotaustralin in etiolated seedlings. Phytochemistry. 1968;7(8):1307‐1312. Bisset F, Clapp R, Coburn R, Ettlinger M, Long Jr L. Cyanogenesis in manioc: Concerning lotaustralin. Phytochemistry. 1969;8(11):2235‐2247.*

11, It is necessary to verify the function of these non-synonymous mutations on gene function, and the function of mutations in promoters on the gene expression level.

*Answer: Thank you very much for your valuable comments.*

*We agree with your opinion and performed a dual luciferase assay to determine the effect of allele variations (at −661 bp upstream of MeMYB4) on the activity of MeMYB4 promoters. The results indicated that the promoter regions of MeMYB4 carrying C had higher activity than those carrying T (Fig. 4H), supporting that those allelic variations affect the expression of MeMYB4 (Lines 305‐309). Meantime, three genes (including Me3GT, UGT85K4, and UGT85K5) were selected to performed virus‐induced gene silencing (VIGS) experiments for their functional validation. However, this job has significantly been delayed due to the serious influence of COVID‐19 in our province. We believe that this concern can be resolved in the next round of review.*

12, Fig. 4C, the phenotypic data described in the figure needs to be shown in the supplementary tables.

*Answer: Thank you very much for your valuable comments. The phenotypic data in Fig. 4C were added in Table. S1.*

13, Figure 4. For the function of MeMYB4, it is really very strange. Because MYB4 belongs to the subgroup 4 of MYB TFs, which are the repressors on phenylalanine metabolism, how can you explain your results conflict with previous reports? The author mentioned MeMYB4 could positively control cyanidin 3-O-glucoside and delphinidin 3-O-rutinoside content via interaction with the promoter of MeCHI and MeF3H. However, the expression level of MeCHI and MeF3H in MeMYB4 transient silencing lines should be added to further prove this suggestion.

*Answer: Thank you very much for your valuable comments. We agree with your opinion that MYB4s are usually the repressors on phenylalanine metabolism. There still are a few reports showing that MYB4s (e.g., NtMYB4a and BjMYB4) act as activators involved in anthocyanin synthesis (Luo et al. 2020; Xie et al. 2014). Of which, NtMYB4a shows 44% protein sequence similarity to MeMYB4 (Fig. 4G).*

*We further did a dual luciferase assay to evaluate the activity of MeMYB4 promoter with different variations (at −661 bp upstream of MeMYB4). The promoter regions of MeMYB4 carrying C had higher activity than those carrying T, in accord with that accessions carrying C had higher content of anthocyanin‐related metabolites than those carrying T (Fig. 4H‐J). As suggested, the expression of MeCHI and MeF3H in MeMYB4 transient silencing lines was added in Fig. 4R. The results showed that expression levels of MeCHI and MeF3H were lower in MeMYB4 transient silencing lines than that in the control plants. These results support the positive role of MeMYB4 in anthocyanin biosynthesis (Lines 319).*

*The above contents were discussed in Lines 507‐509 in the manuscript.*

*References: Luo et al. 2020, Isolation and molecular characterization of NtMYB4a, a putative transcription activation factor involved in anthocyanin synthesis in tobacco, Gene, 760: 144990. Xie et al. 2014, Accumulation and molecular regulation of anthocyanin in purple tumorous stem mustard (Brassica juncea var. tumida Tsen et Lee), J Agric Food Chem, 62(31):7813‐7821.*

14, There are only average metabolite contents in wild cassava accession. The detailed metabolite profiling of 5 wild cassava accessions should be listed respectively.

*Answer: Thank you very much for your valuable comments. The detailed metabolite profiling of 5 wild cassava accessions were added in Table. S2 and Table. S3.*

15, For the figure 5 about the domestication story, it is completely not reliable due to only 5 wild accessions analyzed.

*Answer: Thank you for your criticism. The identification of wild progenitors of cassava was formally reported until 2016 (Bredeson et al. 2016), so the collection of wild progenitors lagged behind. We agree with your opinion that 5 wild accessions were not enough for the domestication analysis, therefore, in this work,*

*1) We collected resequencing data of other 14 wild accessions from the public database (Bredeson et al. 2016; Ramu et al. 2017) and used them for nucleotide diversity analysis (Fig. 5P) and allele frequency analysis (Fig. 5Q). In total, 19 wild accessions were used for this part. These results showed that nucleotide diversity (Pi) around the MeFLS1 region wassignificantly lower in cultivars than in wild accessions, indicating that the MeFLS1 region was under selection during cassava domestication. Moreover, the frequency of the G‐allele was higher in cultivars compared with wild accessions.*

*2) We constructed a phylogenic tree and found that the five wild accessions were distributed in two different branches with other wild accessions (Fig. S7), supporting a representative of the five wild accessions.*

*3) Because of the small sample size of wild accessions, we repeated 10 times of the identification process of metabolites differentially accumulated between wild accessions and cultivars. If a metabolite was found to have differentially accumulated in all of the 10 comparisons, it was considered associated with cassava domestication (see the details in the section “Identification of metabolites associated with domestication”). We applied such a strict statistical method to ensure the reliability of domestication‐associated metabolites.*

*4) We revised our conclusions for the domestication by using the words such as likely/might (Line 121 and Line 386), and added a statement in the discussion (Lines 540‐542): “In the near future, more wild accessions should be collected and sequenced to further verify the accuracy of our domestication analysis because of the limited number of wild accessions used in this work”. We hope you can understand our difficulties to acquire more wild accessions of cassava germplasm.*

*References: Ramu P, Esuma W, Kawuki R, Rabbi IY, Egesi C, Bredeson JV, Bart RS, Verma J, Buckler ES, Lu F. Cassava haplotype map highlights fixation of deleterious mutations during clonal propagation. Nat Genet. 2017, 49(6):959‐963.*

*Bredeson JV, LyonsJB, Prochnik SE, Wu GA, Ha CM, Edsinger‐Gonzales E, Grimwood J, Schmutz J, Rabbi IY, Egesi C, et al. Sequencing wild and cultivated cassava and related species reveals extensive interspecific hybridization and genetic diversity. Nat Biotechnol. 2016, 34(5): 562‐ 570.*

16, To provide insights into the metabolic changes that have taken place during cassava domestication, the authors compared metabolite profiles between 299 cultivated and five representative wild cassava accessions, and identified 132 annotated differentially accumulated metabolites (DAMs), setting the variable importance in projection (VIP) ≥1 and |fold-change| ≥1.5. However, I noted that many metabolites in the VIP didn't meet this criterion in Table S5.

*Answer: Thank you for your valuable comments. In our manuscript, five of the 299 cultivars were randomly chosen and compared with the five wild accessions to identify differentially accumulated metabolites (DAMs). This process was repeated 10 times, and those metabolites differentially accumulated in all of the 10 comparisons were considered associated with cassava domestication.*

*In Table. S5, the statistics were compared between five wild accessions and 299 cultivars. We are sorry for the inappropriate displaying of data. Now Table. S5 and the related contents have been revised accordingly in Table. S13.*

17, Line 303, base on the mGWAS and pGWAS co-locations, the authors revealed that mGWAS signals for 16 flavones were co-located with the pGWAS signal for SR weight per plant on Chr2:12.84 Mb. Please point out these 16 flavones and show the mGWAS of them in figure.

*Answer: Thank you for your valuable comments. We added these 16 flavones and their mGWAS plots in Figure. S8.*

18, Line 312, "The expression of MeFLS1 coincided with the changes in quercetin 3-O-glucoside contents among different tissues". The expression of MeFLS1 was extremely low in S1-S7, while quercetin 3-O-glucoside accumulated in SR according to Fig. 5L and 5M, which didn't support the conclusion. Moreover, the data presented in Fig. 5M were not consistent with Table S6.

*Answer: Thank you very much for your valuable comments. We agree with your opinion that MeFLS1 expressed extremely low in S1‐S7, which may not be good to conclude its correlation with quercetin 3‐O‐glucoside accumulated in SR. These expression data were derived from a single cultivar (SC205). Subsequently, we examined the expression of MeFLS1 and the content of quercetin 3‐O‐glucoside in SR of 22 cassava cultivars, and found that they are significantly correlated (Fig. 5L). This newly generated sub‐figure is used to replace the original Fig. 5L‐M, and the related contents were revised in the manuscript accordingly (Lines 366‐368).*

*We are sorry for the unclear description of Table S6 and Fig. 5M. Table S6 (Table. S14 in the revised manuscript) isthe metabolic content in seven developmentalstages(S1–S7) ofstorage root, the data is log2‐transformed in Fig. 5M (this original sub‐figure has been removed in the revised version).*

19, In Fig. 5O, P=0.08 cannot indicate a significant difference between them. The method of significance test should be described in figure legends.

*Answer: Thank you very much for your valuable comments. The significance was determined by the Student's t‐test, and the contents were revised in the figure legends accordingly.*

20, Improper citation, eg, Line 335.

*Answer: Thank you very much for your valuable comments. This citation is changed to ‘Table S2 and Table S3’.*

21, There is description of the principal component analysis in the section Methods, but is not relevant results in the manuscript.

*Answer: Thank you for your valuable comments. The description of principal component analysis was removed from the section Methods.*

**Second round of review**

**Reviewer 1**

All my comments and suggestions have been answered.

**Reviewer 2**

Although the authors have provided revised manuscript and the quality of the manuscript has been improved, there are several points that need clarifications.

1, Fig. 2G Whether the amino acid non-synonymous mutation caused by the SNP in Me3GT is located in the core functional domain of the protein, and whether it will affect the function of Me3GT. Please use specific data to show the impact of amino acid mutations on the 3D structure of proteins, such as protein surface charge distribution or hydrophobicity. And why changes in the 3D structure of the protein affect the expression of the gene at the transcriptional level. The results of experiments to verify the function of candidate genes in vivo such as Me3GT should be exhibited as soon as possible.

2. In Fig. 3B, the logP of SNP 12:35620552 didn't exceed to the significant threshold. How the causative SNPs was determined?

3. Fig. 4A-C The mGWAS results of Cyanidin 3-O-glucoside and Delphinidin 3-O-rutinoside and the SR endothelium color pGWAS results both show that there are strong association sites in Chr1 and Chr2. Why did the author only analyze the strong signal sites in Chr2? Are there some important genes related to anthocyanin synthesis at the strong signal site in Chr1? Significance test should be conducted in Fig. 4N-P. Please check for similar cases throughout the manuscript. Fig. 4K and 5Q, the number of each group tested should be marked. This should be described in other similar figures and supplementary figures.

4, Fig. 5A, How to cluster cultivated cassava based on metabolite profiles in SR. Please add the clustering method to the article. Fig. 5P, In addition to the Nucleotide diversity (Pi), is there any other analysis that support MeFLS1 being under selection.

5, How the seven periods of cassava SR are divided is not indicated in the RNA‐seq and data analysis. Is there any pictures to show each periods of cassava SR or any standards could be demonstrated?

6, In Table S13, the statistics were compared between five wild accessions and five randomly selected cultivars. Could the five randomly selected cultivated species represent the cultivated population?

7, Line224 The mGWAS signal for the linamarin content of Chr12 is not the strongest and not significant, why this region was chosen. Are there other candidate genes in the Chr16 region?

8, In line 263-269: one of mGWAS signals was detected on Chr2-7.16 Mb, and also were co-located with the GWAS signals for SR endothelium color. Notably, candidate Sc02g007130 (MeMYB4) and possible causative SNP 2:6158256 are over 1Mb away from the lead SNP.

9, Line319, LUC experiments should be added to demonstrate whether the change in priming ability is due to allelic variation in the MeFLS1 promoter region.

10, We noticed the authors supplemented new data, thus a phylogenic tree contained other 14 wild accessions should be constructed to support the domestication process.

11, In the section of Identification of metabolites associated with domestication, detailed method is required: (1) cluster of accessions groups; (2) pi calculation of parameter and software.

12, Sc02g015220(MeFLS1) is detected associated with 16 flavones, our first review attached this question, also still lowly expressed in most of accessions (FPKM<2). What is the result of the expression level of the gene among different variant types?

13, The conclusion of UGT85K5, CYP79D1 and CYP71E7b highly co-expressed is farfetched according to the expression correlation in 22 accessions.

14, Potato genome research found the genes IT1 and SP6A involved in potato tuber formation, and whether there are similar genes contributed to storage root formation in cassava. Is there a convergent evolution of stem enlargement and root enlargement in cassava and potato?

**Authors Response**

**Point-by-point responses to the reviewers’ comments:**

Reviewer #1:

All my comments and suggestions have been answered.

*Answer: Thank you very much for your comments and suggestions, which helped us a lot to improve the quality of our manuscript.*

Reviewer #2:

Although the authors have provided revised manuscript and the quality of the manuscript has been improved, there are several points that need clarifications.

1, Fig. 2G Whether the amino acid non-synonymous mutation caused by the SNP in Me3GT is located in the core functional domain of the protein, and whether it will affect the function of Me3GT. Please use specific data to show the impact of amino acid mutations on the 3D structure of proteins, such as protein surface charge distribution or hydrophobicity. And why changes in the 3D structure of the protein affect the expression of the gene at the transcriptional level. The results of experiments to verify the function of candidate genes in vivo such as Me3GT should be exhibited as soon as possible.

*Answer: Thank you very much for your valuable comments.*

*1) We examined the basic physical and chemical parameters for the proteins of mutants and wild-type by the ProtParam tool, and found that the grand average of hydropathicity (GRAVY) varied among them and the isoelectric point was changed between G80D and the wild-type (Reply Table. S1). These results indicate the possible impact of amino acid mutations on the 3D structure of Me3GT proteins.*

*2) Usually changes in the 3D structure of a protein will not affect the expression of its corresponding gene at the transcriptional level. Here, what we want to say is that changes in the 3D structure of the protein (Me3GT) can affect its enzymatic activities and thereby affect the levels of metabolites (e.g., 8-C-hexosyl-kaempferol O-hexoside). Therefore, we examined the enzymatic activities among six mutants (A6T, G80D, S101C, T145A, S146G, P334S) and allele1 and allele2. Allele2 displayed significant higher enzymatic activity and 8-C-hexosylkaempferol O-hexoside content than allele1 (Fig. 2I-K). Site-directed mutagenesis showed that five of the six nonsynonymous mutations (except S146G) strongly affected the enzymatic activity of Me3GT (Fig. 2K), suggesting that they could be functional SNPs of this gene.*

*3) The CsCMV-CM (cassava common mosaic virus isolate CM) based VIGS system was used to verify the function of Me3GT and UGT85K5. Transient silencing of Me3GT resulted in dramatically decreases in 8-C-hexosyl-kaempferol O-hexoside contents (Fig. 2F-G). Transient silencing of UGT85K5 resulted in significantly decreases in linamarin contents (Fig. 3E-F). These results were revised accordingly in the manuscript.*

2. In Fig. 3B, the logP of SNP 12:35620552 didn't exceed to the significant threshold. How the causative SNPs was determined?

*Answer: Thank you very much for your valuable comments.*

*In Fig. 3B, there was only one clear mGWAS signal (Chr16:32817362) for linamarin content. However, no candidate genes relevant to CG metabolism were obviously found at this locus according to the genomic annotation. Therefore, we began to look at the signals with Pvalues slightly lower than the significant threshold.*

*We identified the candidate genes by the approaches described in the section of “Mining of candidate genes and causative SNPs” (Lines 582-592). We found that the mGWAS signal (SNP 12:35620552) on Chr12 harbors a gene cluster containing five CG biosynthesis genes (CYP79D1, CYP71E7a, CYP71E7b, UGT85K4, and UGT85K5) in a 140-kb region. Moreover, UGT85K5 was highly co-expressed with CYP79D1 and CYP71E7b (Fig. 3D), implying their common involvement in CG biosynthesis. Then, the SNPs located within, upstream, downstream of these three genes were used to examine their associations with the linamarin content, and the significant SNPs were considered as possible causative SNPs.*

3. Fig. 4A-C The mGWAS results of Cyanidin 3-O-glucoside and Delphinidin 3-O-rutinoside and the SR endothelium color pGWAS results both show that there are strong association sites in Chr1 and Chr2. Why did the author only analyze the strong signal sites in Chr2? Are there some important genes related to anthocyanin synthesis at the strong signal site in Chr1? Significance test should be conducted in Fig. 4N-P. Please check for similar cases throughout the manuscript. Fig. 4K and 5Q, the number of each group tested should be marked. This should be described in other similar figures and supplementary figures.

*Answer: Thank you very much for your valuable comments. We have carefully examined the genes around the mGWAS site Chr1-9.13 Mb, however, no genes related to anthocyanin synthesis were obviously found according to the genomic annotation. In contrast, a candidate gene MeMYB4, encoding an ortholog of AtMYB4 involved in anthocyanin biosynthesis, was found around Chr2-6.09 Mb. Therefore, MeMYB4 was selected as the candidate gene to verify its roles in anthocyanin synthesis. According to your suggestion, a significance test was conducted in Fig. 4N-P, and the number of each group tested in Fig. 4K and Fig. 5Q was marked. Similar cases were also checked in other (supplementary) figures.*

4, Fig. 5A, How to cluster cultivated cassava based on metabolite profiles in SR. Please add the clustering method to the article. Fig. 5P, In addition to the Nucleotide diversity (Pi), is there any other analysis that support MeFLS1 being under selection.

*Answer: Thank you very much for your valuable comments. In Fig. 5A, the metabolic data generated from 299 cultivated cassava accessions were log2-transformed to construct a neighbor-joining tree by the software PHYLIP v3.69 and then visualized by the software MEGA6. These contents have added in the manuscript in Lines 613-617.*

*In addition to the nucleotide diversity (Pi), Tajima’D values were calculated between 19 wild and 299 cultivated cassava accessions. The Tajima’D values around the MeFLS1 region were significantly higher in cultivars than in wild accessions (Reply Fig. 1), also supporting that the MeFLS1 region was under selection during cassava domestication.*

5, How the seven periods of cassava SR are divided is not indicated in the RNA‐seq and data analysis. Is there any pictures to show each periods of cassava SR or any standards could be demonstrated?

*Answer: Thank you very much for your valuable comments. Cassava roots were harvested every 40 d from 100 d after planting (DAP) to 340 DAP for a total of seven time-points, referred to as S1–S7 (Ding et al. 2020). Below we also attached the pictures to show each period of cassava SR (Reply Fig. 2). These contents were added in Lines 629-631 in the section of “RNA-seq and data analysis” as suggested.*

*References:*

*Ding Z, Fu L, Tie W, Yan Y, Wu C, Dai J, Zhang J, Hu W. Highly dynamic, coordinated, and stage-specific profiles are revealed by a multi-omics integrative analysis during tuberous root development in cassava. J Exp Bot, 2020, 71(22):7003-7017.*

6, In Table S13, the statistics were compared between five wild accessions and five randomly selected cultivars. Could the five randomly selected cultivated species represent the cultivated population?

*Answer: Thank you very much for your valuable comments. Differentially accumulated metabolites (DAMs) were compared between five wild accessions and five randomly selected cultivars. This process was repeated 10 times, and those metabolites differentially accumulated in all of the 10 comparisons were considered associated with cassava domestication (Table S13). We think these results, to a very large extent, can represent the DAMs between wild and cultivated cassava accessions.*

*Accordingly, we have revised the note of Table S13 as: “Note: The metabolites were compared between five wild accessions and five randomly selected cultivars, and this process was repeated 10 times”.*

7, Line224 The mGWAS signal for the linamarin content of Chr12 is not the strongest and not significant, why this region was chosen. Are there other candidate genes in the Chr16 region?

*Answer: Thank you very much for your valuable comments. There was only one clear mGWAS signal (Chr16:32817362, Fig. 3B) for linamarin content. However, no candidate genes relevant to CG metabolism were obviously found at this locus according to the genomic annotation. Therefore, we began to look at the signals with P-values slightly lower than the significant threshold.*

*We identified the candidate genes by the approaches described in the section of “Mining of candidate genes and causative SNPs” (Lines 582-592). We found that the mGWAS signal (SNP 12:35620552) on Chr12 harbors a gene cluster containing five CG biosynthesis genes (CYP79D1, CYP71E7a, CYP71E7b, UGT85K4, and UGT85K5) in a 140-kb region. Moreover, UGT85K5 was highly co-expressed with CYP79D1 and CYP71E7b (Fig. 3D), implying their common involvement in CG biosynthesis. Then, the roles of UGT85K5 in CG metabolism were examined by in vitro enzyme assays and in vivo gene silencing analyses. That’s why this region was chosen for further analysis.*

8, In line 263-269: one of mGWAS signals was detected on Chr2-7.16 Mb, and also were co-located with the GWAS signals for SR endothelium color. Notably, candidate Sc02g007130 (MeMYB4) and possible causative SNP 2:6158256 are over 1Mb away from the lead SNP.

*Answer: Thank you very much for your valuable comments. We are sorry for our unclear description. Chr2-7.16 Mb is the most significant signal (with the lowest p-value on Chr2) associated with cyanidin 3-O-glucoside, delphinidin 3-O-rutinoside, and SR endothelium color. Besides, there are 17 lead SNPs (from 5.61 to 7.38 Mb) significantly associated with cyanidin 3-O-glucoside, delphinidin 3-O-rutinoside, and SR endothelium color. The candidate genes around these lead SNPs are examined, respectively, according to their functional annotations. The candidate Sc02g007130 (MeMYB4) and the causative SNP 2:6158256 are mined based on the lead SNP 2:6089302 (Table S7), which is 68.95 kb away from the causative SNP. Now we revised “Chr2-7.16 Mb” to “Chr2-6.09 Mb” to eliminate this error.*

9, Line319, LUC experiments should be added to demonstrate whether the change in priming ability is due to allelic variation in the MeFLS1 promoter region.

*Answer: Thank you very much for your valuable comments. We agree with your opinion and performed a dual luciferase assay to determine the effect of allele variations (at −446 bp upstream of MeFLS1) on the activity of MeFLS1 promoters. The results indicated that the promoter region of MeFLS1 carrying G-allele had lower activity than those carrying T-allele (Fig. 5P), suggesting that this allelic variation affects MeFLS1 expression (Lines 343-346).*

10, We noticed the authors supplemented new data, thus a phylogenic tree contained other 14 wild accessions should be constructed to support the domestication process.

*Answer: Thank you very much for your valuable comments. The phylogenic tree (containing other 14 wild accessions) have been revised in Supplementary Fig. S8. The wild and cultivated cassava accessions were highlighted by pink and brown, respectively.*

11, In the section of Identification of metabolites associated with domestication, detailed method is required: (1) cluster of accessions groups; (2) pi calculation of parameter and software.

*Answer: Thank you very much for your valuable comments. Phylogeny of wild and cultivated cassava accessions was generated by the neighbor-joining tree method with genome-wide SNPs (Hu et al., 2021). Nucleotide diversity (Pi) between the wild and cultivated cassava accessions were calculated by VCFtools software in 80-kb sliding windows with 10-kb steps. These contents were added in the manuscript in Lines 600-603.*

12, Sc02g015220(MeFLS1) is detected associated with 16 flavones, our first review attached this question, also still lowly expressed in most of accessions (FPKM<2). What is the result of the expression level of the gene among different variant types?

*Answer: Thank you very much for your valuable comments. We examined the expression levels of MeFLS1 among different variant types and found that accessions carrying G-allele had significantly (P = 0.0003) lower expression level than did the accessions containing Tallele, in accord with the changes of quercetin 3-O-glucoside content (Fig. 5M-N). These contents were revised accordingly in the manuscript in Lines 339-343.*

13, The conclusion of UGT85K5, CYP79D1 and CYP71E7b highly co-expressed is farfetched according to the expression correlation in 22 accessions.

*Answer: Thank you for your criticism. We have collected 465 RNA-seq data in our previous co-expression network analysis (Fu et al. 2021). These datasets were derived from 11 different tissues, 9 biotic and abiotic stresses, and 9 and 7 developmental stages of leaf and root, respectively, thus they represented an extensive and valuable transcriptomic resource for cassava. Using these large-scale datasets, we found that UGT85K5, CYP79D1 and CYP71E7b are also highly co-expressed (Additional file 1: Fig. S7). We hope your concerns have satisfactorily resolved now.*

*References:*

*Fu L, Ding Z, Tie W, Yan Y, Hu W, Zhang J. Large-scale RNAseq analysis reveals new insights into the key genes and regulatory networks of anthocyanin biosynthesis during development and stress in cassava. Ind Crop Prod, 2021, 169: 113627.*

14, Potato genome research found the genes IT1 and SP6A involved in potato tuber formation, and whether there are similar genes contributed to storage root formation in cassava. Is there a convergent evolution of stem enlargement and root enlargement in cassava and potato?

*Answer: Thank you very much for your valuable suggestions. Although studies of the initiation and development of cassava storage root have accumulated over time (Yang et al. 2011; Sojikul et al. 2015; Ding et al. 2020), the key genes involved in cassava storage root formation are still poorly understood. Convergent phenotypic evolution is often based on molecular changes in orthologous genes or pathways (Lenser and Theiben 2013). Therefore, we searched the orthologous genes of IT1 and SP6A in the cassava genome, since SP6A and IT1 are two crucial genes acting as a protein complex in regulating potato tuber formation (Tang et al. 2022). There were two orthologous genes (Manes.12G001600 and Manes.13G000800) of SP6A in cassava, and they both nonexpressed across 11 different tissues according to the gene expression atlas (Wilson et al. 2017). Moreover, no orthologous genes of IT1 were found in cassava. It seems that IT1 and SP6A lost their functions during cassava evolution. Collectively, we think there is not a convergent evolution of stem enlargement in potato and root enlargement in cassava, at least not by the interaction of IT1 and SP6A.*

*References: Ding Z, Fu L, Tie W, Yan Y, Wu C, Dai J, Zhang J, Hu W. Highly dynamic, coordinated, and stage-specific profiles are revealed by a multi-omics integrative analysis during tuberous root development in cassava. J Exp Bot, 2020, 71(22):7003-7017.*

*Lenser T, Theiben G. Molecular mechanisms involved in convergent crop domestication. Trends Plant Sci, 2013, 18(12):704-714.*

*Sojikul P, Saithong T, Kalapanulak S, Pisuttinusart N, Limsirichaikul S, Tanaka M, Utsumi Y, Sakurai T, Seki M, Narangajavana J. Genome-wide analysis reveals phytohormone action during cassava storage root initiation. Plant Mol Biol, 2015, 88(6):531-543.*

*Tang D, Jia Y, Zhang J, Li H, Cheng L, Wang P, Bao Z, Liu Z, Feng S, Zhu X, Li D, Zhu G, Wang H, Zhou Y, Zhou Y, Bryan G, Buell C, Zhang C, Huang S. Genome evolution and diversity of wild and cultivated potatoes. Nature, 2022, 606(7914):535-541.*

*Wilson M, Mutka A, Hummel A, Berry J, Chauhan R, Vijayaraghavan A, Taylor N, Voytas D, Chitwood D, Bart R. Gene expression atlas for the food security crop cassava. New Phytol, 2017, 213(4):1632-1641.*

*Yang J, An D, Zhang P. Expression profiling of cassava storage roots reveals an active process of glycolysis/gluconeogenesis. J Integr Plant Biol, 2011, 53(3):193-211.*

**Third round of review**

**Reviewer 1**

All the concerns have been answered.
